# Additive Manufacturing of Customized Flexible Wearable Sensors for Sweat Analysis with Bespoke, Low-Cost Conductive TPU

**DOI:** 10.1021/acsmeasuresciau.5c00095

**Published:** 2025-09-26

**Authors:** Mayane S. Carvalho, Elena Bernalte, Ana C. M. Oliveira, Eduardo M. Richter, Rodrigo A. A. Muñoz, Robert D. Crapnell, Craig E. Banks

**Affiliations:** † Faculty of Science and Engineering, 5289 Manchester Metropolitan University, Dalton Building, Chester Street, Manchester M1 5GD, Great Britain; ‡ Institute of Chemistry, 28119 Federal University of Uberlândia, 38400-902 Uberlândia, Minas Gerais, Brazil

**Keywords:** Additive manufacturing, Thermoplastic polyurethane (TPU), Carbon black, Graphite, Wearable device, Flexible electrodes, Uric acid

## Abstract

The development of wearable technology that is non-invasive, customizable, comfortable for the user, and able to transmit data to healthcare providers from remote and rural locations has the potential to revolutionize the healthcare sector and aligns with key United Nations Sustainable Development Goals. In this work, bespoke conductive filaments based on thermoplastic polyurethane (TPU) incorporating different ratios of carbon black (CB) and graphite (GRT) were developed, for the first time, via melt extrusion for additive manufacturing of flexible, wearable electrochemical sensors. Eight formulations were systematically evaluated in terms of morphology, electrical resistance, wettability, and electrochemical behavior. The hybrid composition containing 20 wt% CB and 20 wt% GRT demonstrated the best balance between conductivity, mechanical flexibility, printability, and electrochemical activity, while producing a 45% saving in material cost. Surface activation through (electro)­chemical treatment and mechanical polishing significantly improved the electroactive surface area and heterogeneous electron transfer rate, especially for GRT-containing electrodes. The optimized electrode exhibited the highest *k*_0_ and *A*_e_ values and was integrated into a fully printed three-electrode wristband for non-invasive detection of uric acid (UA) in artificial sweat. Differential pulse voltammetry enabled reliable detection of UA in the 2.5–100.0 μmol L^–1^ range with a limit of detection of 1.3 μmol L^–1^ and recovery rates up to 99.7%. The sensor also demonstrated high selectivity against typical sweat interferents such as urea, glucose, and tyrosine. These findings support the potential of additively manufactured carbon-based TPU electrodes for application in wearable sensing platforms for real-time biomarker monitoring.

## Graphical abstract

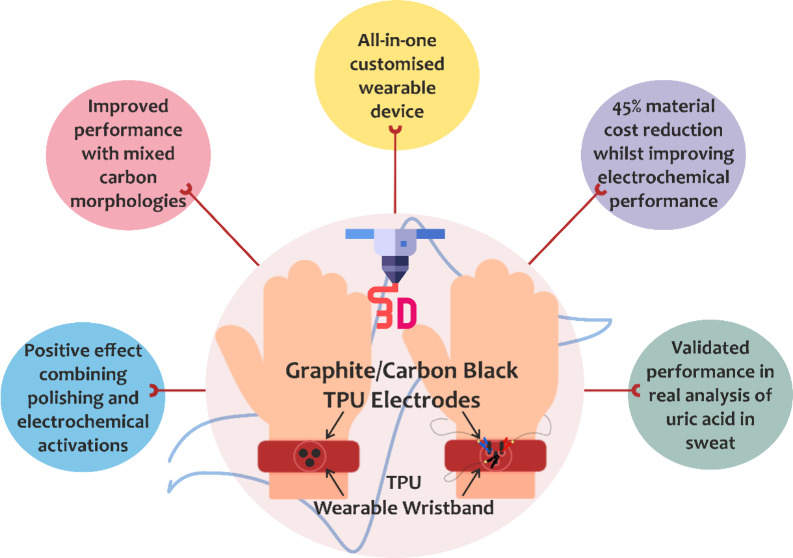

## 1 Introduction

The development of new, bespoke, and reliable wearable sensing technology is at the forefront of innovation that has the potential to curb ever-rising global healthcare costs and effectively transition from reactionary to preventative care.^1–3^ By developing wearable technology that is non-invasive, user-friendly, and capable of transmitting health data to healthcare providers from remote and rural locations, these technologies hold transformative potential to revolutionize the healthcare sector and open new frontiers in medical care. Importantly, these new devices will play a crucial role in meeting and complying with the United Nations Sustainable Development Goals,^4^ in-particular: Goal 3 “Good Health and Wellbeing” through enhancing health outcomes via early detection, chronic disease management, and personalized care; Goal 9 “Industry, Innovation and Infrastructure” through driving medical technology innovation that supports the development of smart healthcare infrastructures; Goal 10 “Reduced Inequalities” through improving healthcare access for marginalized and rural populations; Goal 12 “Responsible Consumption and Production” through promoting sustainable healthcare and reducing the frequency of hospital visits; and Goal 13 “Climate Action” by indirectly helping to reduce the carbon footprint of healthcare systems.

Two synergetic methodologies that can significantly aid the rapid development of these systems are electrochemistry and additive manufacturing. Electrochemical sensors have been synonymous with wearable sensors within the literature over the past decade due to their design flexibility, availability of low-cost portable potentiostats, rapid analytical nature, and reliability.^5–8^ More recently, the symbiosis between electrochemistry and additive manufacturing techniques, such as fused filament fabrication (FFF), has significantly expanded the design possibilities for electrochemical devices, enabling accessible and adaptable sensor production.^9^ This manufacturing flexibility is particularly valuable in the development of wearable systems, where low cost, structural customization, and mechanical resilience are crucial features.^10^

The rapid rise in additive manufacturing electrochemistry has stemmed from the availability of low-cost commercial 3D-printers and electrically conductive filaments. This allowed researchers to explore the design flexibility additive manufacturing could offer through the development of various shaped electrodes,^11^ lab accessories,^12^ and electrochemical equipment.^13–16^ Although functional, these systems are significantly inferior to classical electrodes due to the poor conductivity of the filament, and therefore researchers have explored various methods to improve the performance through “activation” of the electrodes,^17–23^ with the most commonly used being electrochemical^24^ and mechanical^21^ activations. Although improvements were seen, these systems were still subpar due to the quality of the conductive filament and as such, researchers have extensively explored the production of their own bespoke filaments.^25^ Initially, they focused on the development of poly­(lactic acid) (PLA) based filaments with single^26–28^ or mixed carbon morphologies,^29–33^ which has now progressed to the inclusion of functional fillers^34,35^ or metallic nanoparticles.^36–38^ Carbon black (CB) and graphite (GRT) are often selected as conductive fillers in composite filaments because of their ability to support charge transport and maintain structural integrity when combined with thermoplastic binders.^29,39^ Although now producing electrochemical performance worthy of being a mainstay of the electrochemist’s arsenal, these PLA-based systems still suffer from the fallibilities of the polymer, such as poor chemical stability^40^ and solution ingress,^41^ making them essentially single use items.

To address these limitations, the choice of matrix polymer plays a fundamental role, with researchers next successfully exploring poly­(ethylene terephthalate glycol) (PETg)^42,43^ and poly­(propylene) (PP)^44,45^ alternatives. Thermoplastic polyurethane (TPU) stands out when considering wearable applications as it combines excellent elastic deformation capacity with ease of processing, offering both durability and softness, which are essential properties for the fabrication of wearable and deformable sensor platforms.^46^ Unlike rigid polymers such as PLA or PETg, TPU enables dynamic mechanical adaptability, supporting body movement without compromising electrode integrity.^47^ Additionally, TPU’s compatibility with a range of carbon-based fillers facilitates the formation of conductive pathways when processing conditions and formulations are optimized.^48^ However, to improve the electrochemical response of printed electrodes, especially from more durable polymers, surface conditioning strategies or “activation” mentioned previously are still required, including abrasive finishing and electrochemical exposure to alkaline solutions to reveal active sites and increase conductivity. Studies have shown that such postprinting activation can substantially enhance voltammetric performance and sensitivity, including for TPU.^9,24,31,48^

In this study, a series of CB/GRT/TPU composite filaments were developed for the fabrication of flexible electrochemical sensors using additive manufacturing. The additive manufactured electrodes produced using those filaments underwent surface treatments combining mechanical polishing and (electro)­chemical activation to optimize their electrochemical performance. Following this, to provide proof-of-concept for the synergy between additive manufacturing and electrochemistry to produce the next-generation of wearable sensors, the bespoke conductive TPU was incorporated into a TPU-wristband and applied to detect uric acid in synthetic sweat. This work demonstrates the potential of TPU-based conductive filaments for wearable applications and highlights how surface treatment protocols and material composition affect the analytical performance of printed electrodes.

## 2 Experimental Section

### 2.1 Chemicals and Solutions

All chemicals used in this study were of analytical grade and used as received, without further purification. All aqueous solutions were prepared using deionized water with a resistivity not less than 18.2 MΩ·cm, obtained from a Milli-Q Integral 3 system (Millipore, Watford, UK). Sodium chloride (≥99.0%), graphite powder (<20 μm), hexaammineruthenium­(III) chloride ( ≥98%), potassium ferricyanide (≥99%), potassium ferrocyanide (≥98.5–102%), sodium hydroxide (≥98%), potassium chloride (≥99.0–100.5%), potassium dihydrogen phosphate (≥99%), dipotassium hydrogen phosphate trihydrate (≥99%), lactic acid (≥85.0%), calcium chloride dihydrate (≥99.0%), l-tyrosine (≥98.0%), d-glucose (≥99.0%) and urea (≥98%) were obtained from Merck (Gillingham, UK). Uric acid (UA) was obtained from Fisher Scientific (Loughborough, UK). Carbon black (CB) was sourced from PI-KEM (Tamworth, UK), and thermoplastic polyurethane (TPU, Desmopan 3855) was purchased from Hardie Polymers (Glasgow, UK).

The phosphate-buffered saline (PBS) supporting electrolyte was prepared at a concentration of 0.1 mol L^–1^ and pH 7.4. Stock solutions of uric acid (UA) were freshly prepared in PBS by sonication for 5 min to ensure complete dissolution.

Artificial sweat was prepared according to previously reported protocols,^49,50^ using sodium chloride (NaCl), potassium chloride (KCl), calcium chloride dihydrate (CaCl_2_·2H_2_O), lactic acid (C_3_H_6_O_3_), and urea (CH_4_N_2_O). All components were freshly dissolved in deionized water and subsequently diluted in a 1:1 ratio with phosphate-buffered saline (PBS, 0.1 mol L^–1^, pH 7.4) before electrochemical analysis.

### 2.2 Filament Production

To produce the conductive filaments used in this study, composite materials containing 40 wt% of total conductive phase and 60 wt% of thermoplastic polyurethane (TPU) were formulated. This proportion was selected based on prior optimization studies carried out by Oliveira et al.^48^ The conductive phase was composed of carbon black (CB) and graphite (GRT), combined in varying ratios across eight formulations (40 wt% CB to 5 wt% CB and 35 wt% GRT). Before processing, the TPU granules were dried at 110 °C for 24 h to eliminate any residual moisture content. The weighed amounts of TPU and carbon fillers were manually premixed before being transferred into the 63 cm^3^ chamber of the Thermo Haake Rheomix 600 internal mixer. This manual premixing step ensured preliminary homogenization and reduced the risk of filler segregation, facilitating a more uniform dispersion during melt compounding. Mixing was performed at 210 °C and 70 rpm for 5 min using a Thermo Haake Rheomix 600 internal mixer with Banbury rotors (Thermo-Haake, Germany), coupled to a Haake Polydrive control unit. Potential problems at this stage mainly involve residual moisture (leading to processing instabilities) or poor homogenization of CB/GRT, which could result in heterogeneous conductivity. These issues were avoided by the combination of predrying TPU and careful manual premixing prior to melt compounding. Once the mixing process was complete, the hot composites were allowed to cool to room temperature and subsequently granulated into smaller fragments using a Rapid 1528 granulator (Rapid, Sweden), improving feed consistency for the extrusion step. The granulated material was fed into a Filabot EX2 filament extruder (Filabot, Vermont, USA), fitted with a single-screw system and two independent heating zones set to 60 °C and 210 °C, respectively. The molten composite was extruded through a 1.75 mm circular die, passed through an inline air-cooling system (Airpath, Filabot), and continuously wound onto a spool. Finally, the extruded filaments were postdried in a fan-assisted oven at 80 °C overnight to ensure complete removal of residual humidity. The finished filaments were then stored in sealed containers until use in additive manufacturing.

### 2.3 Fabrication of Electrodes and Wearable Wristband via Additive Manufacturing

The “lollipop” style additive manufactured electrodes used for characterization in this work were printed using a custom 3D printer configured with an E3D ToolChanger system. The extrusion system employed Hemera V6 hot-end modules paired with 0.4 mm diameter nozzles. All electrodes were fabricated using a rectilinear infill^51^ pattern to ensure uniform internal structure, with the nozzle and print bed maintained at 238 °C and 50 °C, respectively. To enhance TPU adhesion during printing, the borosilicate glass bed was lightly coated with a standard water-soluble glue stick. The print speed was set at 10 mm/s across all movements in PrusaSlicer. A sequential manufacturing approach was adopted, wherein each electrode was fully printed before initiating the next. Between sequential prints, a cleaning command was incorporated into the G-code to remove residue from the nozzle using built-in brass brushes, maintaining extrusion quality throughout the printing process. These specific printing parameters, including fixed print speed and sequential fabrication, were selected in accordance with previously described protocols in the literature.^48^ The “lollipop” electrodes used for electrochemical characterization followed the CAD dimensions reported in the manuscript (5 mm disc, 2 mm track width; overall nominal thickness ∼ 1 mm), and were fabricated with sequential layers corresponding to this final thickness, ensuring minimal variability between samples.

Two flexible wristband models were also printed using the bespoke TPU filament for the working, counter and reference electrodes, and commercial nonconductive TPU filament (Ninjatek NinjaFlex) for the body of the wristband. The “screw” style electrodes used in this application were 2 mm thick, Ø5.3 mm discs with an 8 mm long cylindrical connection stem of Ø1.8 mm. The wristband was a rectangular design of 225 mm length, 25 mm width, and 2 mm thickness where holes for the three electrodes were incorporated into the design evenly spaced 7 mm apart. Both “screw” electrodes and wristband were printed on a Prusa MK3S+ printer modified with an E3D Revo Roto Extruder and the poly­(ether imide) (PEI) print bed was covered in a thin layer of standard water-soluble adhesive glue prior to printing. All prints were performed with a nozzle temperature of 238 °C, bed temperature of 50 °C, print speed of 10 mm s^–1^, and an infill of 100% and 15% for the electrodes and wristband, respectively. One design of wristband featured a raised ring structure around the electrode region to form a contained electrochemical cell for laboratory testing. The second model served as a wearable prototype for direct application on the forearm. Both wristband designs included specific slots to accommodate the insertion of conductive electrodes produced with the CB/GRT/TPU filaments developed in this work.

### 2.4 Physicochemical Characterization

Surface morphology and structural features of the printed composites were examined by scanning electron microscopy (SEM) using a Crossbeam 350 FIB-SEM system (Carl Zeiss Ltd., Cambridge, UK), equipped with a field emission electron source. Images were acquired using a secondary electron–secondary ion (SESI) detector. Prior to imaging, the samples were mounted on 12 mm aluminum SEM stubs (Agar Scientific, Essex, UK) using conductive carbon adhesive tabs (Agar Scientific), and subsequently coated with a 3 nm gold/palladium (Au/Pd) layer using a Leica EM ACE200 sputter coater.

Thermogravimetric analysis (TGA) was carried out using a Discovery Series SDT 650 instrument operated via Trios software (TA Instruments, Delaware, USA). Samples were placed in 90 μL alumina crucibles and subjected to a temperature ramp of 10 °C per minute, ranging from 0 to 800 °C, under a constant nitrogen flow of 100 mL per minute.

Chemical composition and surface elemental states were analyzed by X-ray Photoelectron Spectroscopy (XPS) using a Kratos AXIS Supra instrument (Kratos Analytical Ltd., UK), operating with a monochromatic Al Kα radiation source (1486.6 eV) at 225 W. Spectra were acquired in fixed transmission mode, with pass energies of 160 eV for wide scans and 20 eV for high-resolution scans. The instrument’s slot mode was applied to yield an analysis area of approximately 700 × 300 μm. The energy scale was calibrated by referencing the C 1s peak of sp^2^-hybridized carbon to 284.5 eV, a common though limited calibration standard.^52^

Contact angle measurements were performed using a custom equipment built in-house by depositing 40 μL droplets of deionized water onto the electrode surfaces using a micropipette. Each droplet was carefully placed to avoid spreading or deformation at impact. Images were captured using a standard USB digital microscope connected to a computer. The contact angles were analyzed using the DropSnake plug-in within ImageJ software.^53^ For each surface condition, three independent measurements were conducted, and the results are presented as the mean value ± standard deviation.

### 2.5 Electrochemical Measurements and Surface Activation of Additive Manufactured Electrodes

All electrochemical measurements were performed at room temperature using an Autolab PGSTAT100N potentiostat (Metrohm Autolab BV, Utrecht, The Netherlands) controlled by NOVA 2.1.7 software. Electrodes were 3D printed in a standard “lollipop” geometry—disk diameter = 5 mm, connection length = 8 mm, width = 2 mm, and overall thickness = 1 mm.^54^ A conventional three-electrode cell was employed, comprising the 3D printed electrode as working electrode, a commercial Ag|AgCl|KCl (3 M) reference electrode, and a nichrome-wire counter electrode. All experiments were carried out in the presence of dissolved oxygen, unless otherwise stated.

Cyclic voltammetry (CV) was first conducted with two benchmark redox probes: 1.0 mmol L^–1^ [Ru­(NH_3_)_6_]^3+^, and 1.0 mmol L^–1^ [Fe­(CN)_6_]^3–/4–^, each prepared in 0.1 mol L^–1^ KCl. Prior to use, the [Ru­(NH_3_)_6_]^3+^ solutions were thoroughly deaerated by bubbling high-purity nitrogen for 10 min to eliminate dissolved oxygen. Voltammograms were used to assess electron-transfer kinetics and peak-to-peak separations.

The electrochemical active surface area (*A*_e_) was estimated from the calculation of the double-layer capacitance (*C*_dl_) using CV in 0.1 mol L^–1^ KCl. Measurements were performed in the potential range of 0.0 to +0.3 V (vs Ag|AgCl|KCl (3 M)) at scan rates from 5 to 30 mV s^–1^. The *C*_dl_ values were calculated from the slope of the linear plot of current difference (Δ*J*) at +0.15 V versus the scan rate, as previously reported in the literature.^55^

Detection of UA was carried out using differential pulse voltammetry (DPV). The DPV parameters were: step potential = 5 mV and pulse amplitude = 50 mV, following the conditions reported by.^56^ Electrodes were evaluated using two setups: one with external commercial reference and counter electrodes, and another with a fully integrated 3D printed three-electrode system composed entirely of CB/GRT/TPU material.

Before use, all electrodes underwent surface conditioning. Two types of treatments were applied. The first was a mechanical polishing (P) procedure performed using 3 M 1200 grit abrasive paper in the presence of deionized water. Abrasion was carried out manually in a “∞” (figure-eight) motion and repeated eight times. This procedure did not significantly reduce the electrode thickness but effectively uniformised the surface, leaving it finely scratched to expose conductive sites and remove superficial residues. The second treatment was an (electro)­chemical activation (EC), conducted in 0.5 mol L^–1^ NaOH solution by applying a potential of +1.4 V for 200 s followed by −1.0 V for 200 s (vs Ag|AgCl|KCl (3 M)). This activation protocol was adapted from procedures previously established in the literature.^24^ After the EC step, the electrodes were thoroughly rinsed with deionized water and dried.

## 3 Results and Discussion

### 3.1 Optimization of CB and GRT Ratios for the Production of Conductive TPU Filaments

The development of conductive filaments is a pivotal step toward enabling reliable additive manufactured electrochemical sensors. In this study, thermoplastic polyurethane (TPU) was selected as the polymer matrix due to its elastomeric properties, which combine softness, durability (in particular excellent abrasion resistance), resistance to oils and grease, and ease of processing—essential features for wearable devices. As conductive fillers, carbon black (CB) and graphite (GRT) were incorporated in varying ratios while keeping the total carbon content fixed at 40 wt%. This is an important strategy for reducing the material cost of devices as seen in previous work,^29,30,44^ a vital parameter for commercial uptake in healthcare practice. This work aims to evaluate the electrical impact of combining spherical, high-surface-area CB particles with the lamellar structure of GRT within a TPU matrix. Table 1 presents the composition, measured diameters, and corresponding electrical resistance of each filament formulation.

**Table 1:** Compositions, Diameters and Electrical Resistance of Conductive Filaments Prepared with Varying Ratios of CB and GRT in a TPU (60 wt%) Matrix

**Filament**	wt% CB	wt% GRT	**Diameter (mm)**	**Resistance per 10 cm (Ω)**
01	40		1.75 ± 0.07	311 ± 5
02	35	05	1.70 ± 0.10	325 ± 8
03	30	10	1.65 ± 0.08	332 ± 10
04	25	15	1.75 ± 0.03	380 ± 17
05	20	20	1.75 ± 0.05	555 ± 20
06	15	25	1.70 ± 0.10	1355 ± 32
07	10	30	1.65 ± 0.04	6150 ± 173
08	05	35	1.65 ± 0.07	28000 ± 640

The results in Table 1 show a clear trend, where the higher the CB content, the lower the electrical resistance. The filament 01 composed exclusively of CB (40 wt%) achieved the best electrical performance, with a resistance of 311 ± 5 Ω across 10 cm of filament. Small substitutions of CB by GRT (e.g., filaments 02 and 03) resulted in modest increases in resistance (325 ± 8 and 332 ± 10 Ω across 10 cm of filament, respectively), suggesting that at low concentrations, GRT does not significantly disrupt the conductive network. However, as the GRT content was increased beyond 15 wt%, resistance values escalated sharply culminating in 28000 ±  640 Ω across 10 cm of filament for the 5 wt% CB/35 wt% GRT formulation (filament 08). This trend is clearly illustrated in Figure S1, where resistance rises exponentially as CB content decreases. This behavior aligns with the percolation threshold model. CB, with its small particle size and high surface area, more effectively forms interconnected conductive paths within the TPU matrix.^57,58^ In contrast, GRT contributes less efficiently to percolation when present in high proportions, likely due to its planar morphology and lower surface contact.^59^ As a result, insufficient CB content compromises the continuity of the conductive network, explaining the drastic increase in resistivity for GRT-dominant filaments.

Beyond electrical performance, the visual and mechanical aspects of the filaments also changed with composition. As shown in Figure S2, filaments with higher GRT content—particularly from the 15 wt% CB/25 wt% GRT formulation onward (Figure S2F–H)—exhibited a moderate but perceptible reduction in flexibility. In contrast, filaments with higher CB content (Figure S2A–E) maintained a more compliant structure. This observation suggests that although GRT imparts rigidity, its effect on flexibility becomes appreciable only at higher concentrations. This factor is particularly relevant for wearable applications, where mechanical adaptability is required without compromising sensor stability.

Notably, the formulation containing 20 wt% CB and 20 wt% GRT (Filament 05) exhibited a moderate resistance of 555 ± 20 Ω per 10 cm of filament, which is consistent with the performance reported for similar carbon-based hybrid systems in the literature. Ferreira et al.^44^ developed polypropylene-based filaments containing 50 wt% of a CB/GRT mixture (1:1), achieving a resistance of 223 ± 12 Ω per 10 cm, while a 75/25 CB/GRT formulation reached an even lower resistance of 177 ± 4 Ω, highlighting the dominant role of CB in establishing conductive pathways. Arantes et al.^29^ reported a resistance of 875 ± 38 Ω in a recycled PLA filament containing 15 wt% CB and 10 wt% GRT, using castor oil as a plasticizer. Additionally, Augusto et al.^30^ evaluated recycled PLA filaments with a fixed total carbon loading of 15 wt% and varying CB:G ratios. Their 60/40 (CB/GRT) formulation exhibited a resistance of 6.9 ± 0.7 kΩ per 10 cm, while the 40/60 (CB/GRT) formulation showed a substantially higher resistance of 18 ± 2 kΩ per 10 cm. These values, although measured under different polymeric conditions and filler loadings, provide further insight into the impact of CB/GRT ratio on electrical performance. Although resistance values vary depending on the polymer type, filler ratio, and processing method, the 20/20 composition developed in this work shows competitive performance relative to literature benchmarks. these results reinforce that a balanced combination of CB and GRT is essential to maintain conductivity while enabling filament printability and structural flexibility.

Visual inspection of the printed electrodes (Figure S3) reveals notable differences in printability as the graphite content increases across the CB/GRT/TPU formulations. While all filaments maintained consistent diameters (1.6–1.7 mm), the shape fidelity and surface uniformity of the printed electrodes were visibly affected by higher graphite loadings. Filaments with up to 20 wt% GRT (Figure S3A–E) produced electrodes with well-defined contours and homogeneous surfaces, confirming good melt flow and layer adhesion during fused filament fabrication. However, at higher GRT contents (Figure S3F–H), print defects became more apparent, including surface roughness, irregular edges, and incomplete layer filling—indicative of impaired extrusion and reduced interlayer fusion. These visual differences reinforce the importance of optimizing the CB/GRT ratio not only for conductivity and flexibility but also for reliable processability and shape definition, which are critical for sensor fabrication via additive manufacturing. This outcome is consistent with the expected influence of graphite morphology, since its lamellar structure can hinder homogeneous extrusion and interlayer fusion when used in higher loadings, thereby compromising print uniformity, which is an essential parameter for reproducible electrode performance.^60^

### 3.2 Physicochemical and Surface Characterization

Characterization of the filaments was performed using thermogravimetric analysis (TGA), as illustrated in Figure S4. This technique provides essential information regarding the impact of incorporating carbon black (CB), as well as the combined addition of graphite (GRT) and CB, on the thermal stability of the TPU-based composite. Furthermore, it allows for verification that the intended proportions of CB and GRT are indeed present in the material. As shown in Figure S4, the results for both filaments were highly comparable. There is no significant shift in the initial degradation temperature in either case, indicating that the presence of CB, whether alone or in combination with GRT, does not compromise the thermal stability of the polymer. Moreover, the initial degradation temperature for both materials is approximately 300 °C, which is considerably higher than the temperature required for filament extrusion. By subtracting the steady-state value after degradation of the virgin TPU pellet from each filament curve, the resulting filler content values confirm that no material loss occurred during the manufacturing process.

As mentioned above, within the field of additive manufacturing electrochemistry it is commonplace to perform surface treatments to the working electrodes to improve the accessibility of the surface, in particular for inner-sphere probes.^17^ The most common of these methods is either electrochemical activation^24^ or mechanical polishing,^21^ with only electrochemical being reported for conductive TPU.^48^ In this work, we aim to optimize the activation procedure and observe the differences obtained by incorporating GRT in addition to CB. As such, for physicochemical characterization of the electrodes, four conditions were studied: untreated, polished (P), electrochemically activated (EC), and polished and electrochemically activated (P-EC).

Then, morphological analysis of the additive manufactured electrodes produced at different CB:GRT ratios, both before and after surface activation, was performed using scanning electron microscopy (SEM), as depicted in Figure 1.

**Figure 1:**
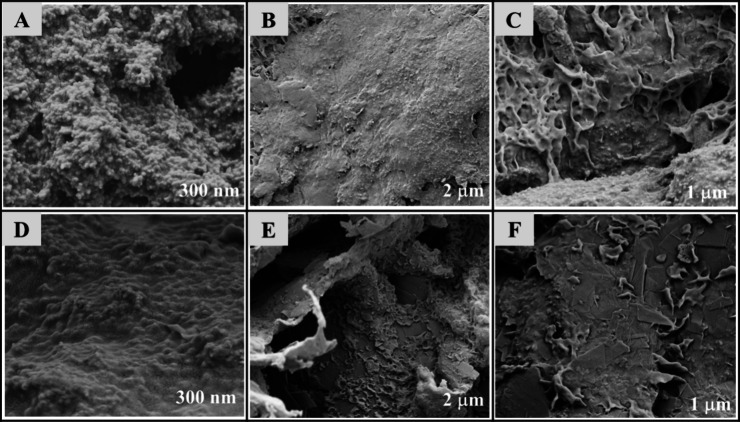
Surface micrograph of CB/TPU and CB/GRT/TPU electrodes under different surface treatments. Images A–C correspond to 40 wt% CB/TPU electrodes in the as-printed, mechanically polished, and combined polishing and (electro)­chemical activation conditions, respectively. Images D–F show 20 wt% CB/20 wt% GRT/TPU electrodes under the same sequence of treatments.

The morphological differences observed between the 40 wt% CB/TPU and 20 wt% CB/20 wt% GRT/TPU electrodes can be directly attributed to the structural nature of the carbon materials used. CB/TPU electrodes (Figure 1A) exhibit visibly rough surfaces with defined 3D-printing layer marks—typical of carbon black’s granular and aggregated morphology, which tends to form point-to-point conductive networks but does not favor interlayer fusion in polymer-based filaments during FFF printing.^25,48,61^ After mechanical polishing (Figure 1B), surface roughness is visibly reduced, yet microdefects remain. Following (electro)­chemical activation (EC) (Figure 1C), a more consistent exposure of CB conductive domains is observed, in agreement with the findings of Barbosa et al.^62^ and Rocha et al.,^63^ who reported that such treatment removes residual polymers and enhances the accessibility of conductive sites in CB-based printed electrodes.

In contrast, CB/GRT/TPU electrodes show a much more homogeneous and compact surface even in the as-printed state (Figure 1D). This is due to graphite’s lamellar structure, which exhibits a high aspect ratio and promotes π–π stacking networks that enhance filament interlocking and reduce void formation—a behavior similarly reported in 3D-printed graphite structures.^64,65^ After mechanical polishing (Figure 1E), this formulation exhibits fewer surface defects and, following the combined P-EC treatment (Figure 1F), reaches an almost ideal surface uniformity. These results indicate that the combination of graphite incorporation with physical and chemical surface treatments ensures an improved morphological integrity,^66^ which strengthens the electrode–electrolyte interface and will be pivotal for reliable sensing applications.

Concomitantly, the surfaces of the electrodes, before and after activation, were also evaluated using X-ray Photoelectron Spectroscopy (XPS). For electrodes containing 40 wt% CB, when fitting the C 1s spectra for as-printed TPU electrodes (CB/TPU; Figure S4B), five symmetric peaks were necessary to achieve an adequate fit. The main peak, located at 285 eV, corresponds to C—C sp^3^ bonding. Additionally, three minor peaks are assigned to C—O, C=O, and O—C=O bonding, which are consistent with the chemical structure of TPU. In contrast, for the polished electrodes (P-CB/TPU; Figure S5B), the electrodes treated with NaOH (EC-CB/TPU; Figure S6B), and those treated with the combined surface treatments (P-EC-CB/TPU; Figure 2B), four symmetric peaks were sufficient for fitting. The main peak remains associated with C—C sp^3^ bonding at 285 eV, while two minor peaks are attributed to C—O and O—C=O groups.

**Figure 2:**
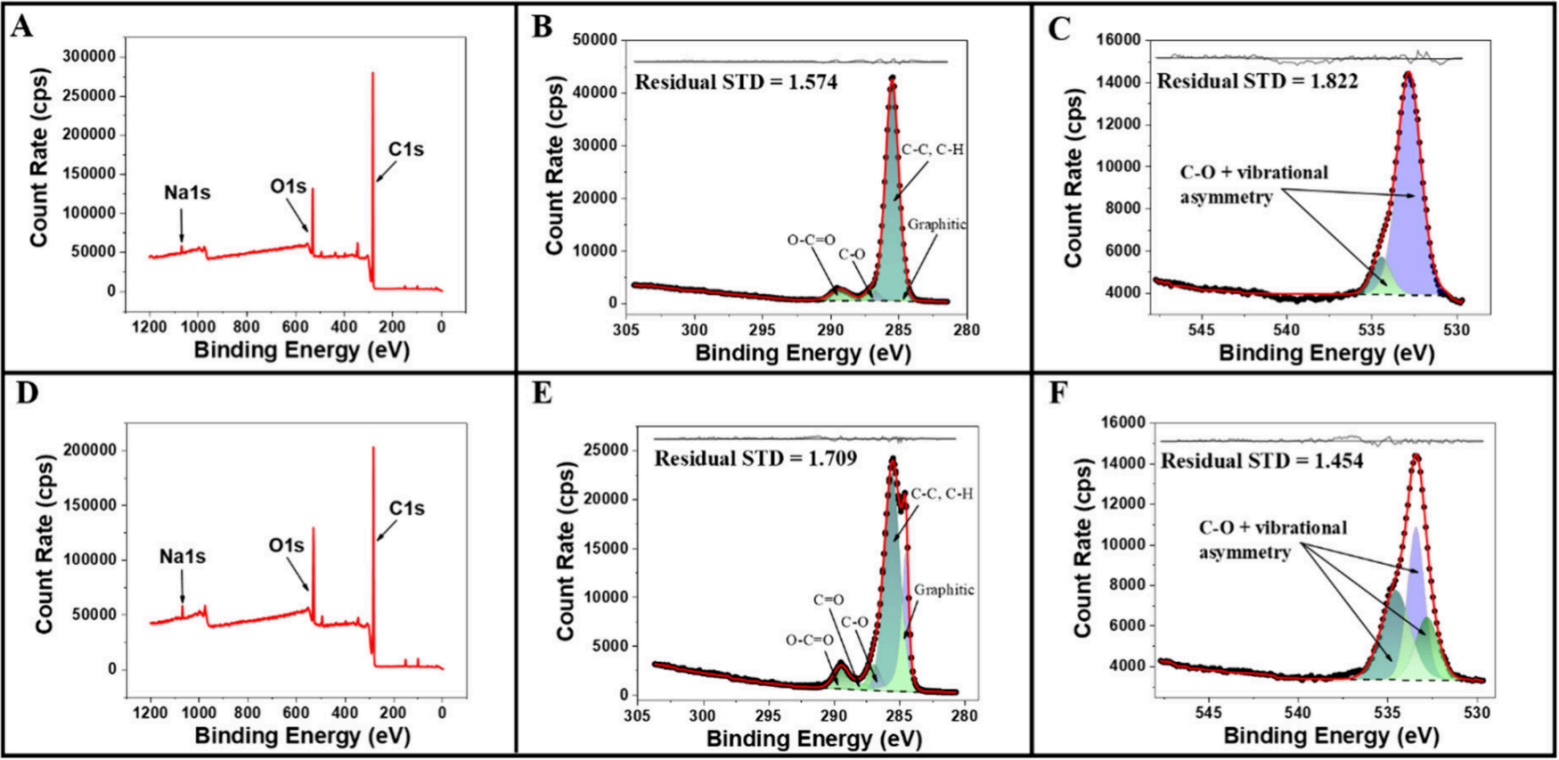
XPS data of the 40CB/TPU filament: A) Survey, B) C 1s and C) O 1s spectra of P-EC-CB/TPU electrode. XPS data of the 20CB/20GRT electrode: D) Survey, E) C 1s and F) O 1s spectra of P-EC-CB/GRT/TPU electrode.

For all samples, an additional asymmetric peak located at 284.5 eV was required, which is attributed to the X-ray photoelectron emission of graphitic carbon. A summary of the peak assignments with their relative atomic concentrations for each sample is presented in Tables S1 and S2. A clear increase in the graphitic peak intensity is observed following surface polishing, both after the printing process and when combined with electrochemical treatment (Table S1). This is attributed to the greater exposure of the conductive material (CB), which contains sp^2^ hybridized C—C bonds within its aromatic planes (carbon rings) and, in some cases, between planes due to the inherent structural disorder of the material. This confirms that the polishing process effectively removes surface TPU, thereby exposing the underlying conductive carbon black.

For the electrodes containing graphite (20CB/20GRT), five symmetric peaks were observed in the absence of surface treatment (Figure S4E), as well as after NaOH treatment (EC-CB/GRT/TPU, Figure S6E) and after the combined treatment (P-EC-CB/GRT/TPU; Figure 2E). These peaks are associated with C—C sp^3^ bonding at 285 eV and minor peaks corresponding to C—O, C=O, and O—C=O, reflecting the TPU structure. In contrast, the electrode treated exclusively with mechanical polishing (P-CB/GRT/TPU, Figure S5E) displays only 4 symmetric peaks. As with the other samples, the presence of the asymmetric graphitic peak is consistently detected. Following the polishing processes, which expose the conductive materials, an increase in the intensity of the graphitic peak is evident. This is expected, given that graphite is a constituent of these electrodes (Table S2). It is also important to mention that the various surface treatments induce changes in the functional groups present on the electrode surface, which may influence their electrochemical performance.

Next, the wettability of the CB/GRT/TPU-based electrodes was investigated via static contact angle measurements, aiming to evaluate the effect of surface treatments on the hydrophilic/hydrophobic character of the printed surfaces. Both the top and bottom sides of the electrodes were analyzed, as fused filament fabrication (FFF) printing typically leads to surface heterogeneity due to differences in extrusion orientation and contact with the build platform.^67^ In this analysis, the designation CB/GRT/TPU refers specifically to electrodes printed from the filament composed of 20 wt% CB, 20 wt% GRT, and TPU. As shown in Table S3, the untreated CB/GRT/TPU electrode exhibited high contact angles (120 ± 2° on the top and 114 ± 10° on the bottom), indicating a predominantly hydrophobic surface, which is consistent with the intrinsic hydrophobic nature of TPU reported in the literature.^68^ Figure S7A,B visually supports this observation with near-spherical droplet profiles on both surfaces. Interestingly, polishing alone (P-CB/GRT/TPU) did not significantly change the contact angles, with values remaining around 120° on both surfaces (Table S3). This suggests that while the abrasive treatment may smooth or expose surface microfeatures, it is insufficient to modify the surface chemistry responsible for water interaction. Electrochemical activation in alkaline medium (EC-CB/GRT/TPU), on the other hand, induced a clear reduction in contact angle on the bottom surface (104 ± 6°), likely due to partial removal of the TPU layer and exposure of oxidized carbon sites with higher surface energy.^24,48^ However, only when mechanical polishing was combined with electrochemical activation (P-EC-CB/GRT/TPU) a substantial decrease was observed, particularly on the top surface, where the contact angle dropped to 99 ± 4°. This shift from hydrophobic to moderately hydrophilic behavior (Figure S7C,D) indicates increased surface roughness and chemical modification, likely involving the incorporation of hydroxyl or carboxyl groups as described in the literature for alkaline activation of carbon materials.^24,69^ These findings demonstrate that the synergistic combination of physical and chemical treatments is more effective in tuning the surface wettability of printed carbon electrodes. This enhancement in hydrophilicity may promote improved electrochemical performance in aqueous media by facilitating analyte diffusion and electrolyte contact with the active surface.

### 3.3 Electrochemical Characterization of TPU Electrodes

#### 3.3.1 Cyclic Voltammetry in [Fe­(CN)_6_]^4–/3-^

To evaluate the effect of these surface treatments on the electrochemical performance of the additive manufactured electrodes, they were tested against the commonly used inner-sphere probe [Fe­(CN)_6_]^4–/3-^ (1 mM in 0.1 M KCl). This cyclic voltammetric data provides key insights into how filler composition and surface treatment influence charge transfer behavior. The cyclic voltammograms obtained at 25 mV s^–1^ for each filament composition and each surface treatment are presented in Figure S8. A clear example of limited performance is observed for the electrode composed of 5 wt% CB and 35 wt% GRT (Figure S8H). This composition presented poorly defined redox peaks and markedly low peak currents across all examined surfaces, on the electrodes as printed. Even after (electro)­chemical and combined polishing treatments, the voltammetric profile remained distorted, with Δ*E*_p_ values exceeding 490 mV and *I*_pa_ under 40 μA (Table S4). This poor behavior is attributed to insufficient percolation pathways, as the high GRT content fails to establish an interconnected conductive network, especially in the presence of a large proportion of insulating TPU. At the same time, it is important to note that, as previously illustrated in Figure S3H, the electrode printed using 35 wt% GRT exhibited poor print quality, which could also limit the electrochemical performance. In contrast, as GRT content was progressively reduced and CB content increased, a clear improvement in redox activity was observed for the electrodes treated by combining polishing and (electro)­chemical treatment (Figure S8A–G).

Overall, it was observed that the untreated electrodes exhibited limited electrochemical activity due to the insulating nature of the TPU surface and the limited exposure of conductive domains. Electrochemical activation alone improved peak definition modestly across most compositions by partially removing the polymer matrix and exposing more electroactive sites. Interestingly, the contributions of the combined polishing and (electro)­chemical (P-EC) treatment were only evident in formulations containing higher graphite content. For instance, the 20CB/20GRT and 15CB/25GRT electrodes showed a significant enhancement in current and Δ*E*_p_ after P-EC treatment. This could be potentially due to the exposure of increased amounts of edge planes within the graphite through abrasion and activation. The 20CB/20GRT electrode (Figure S8E) emerged as the most electrochemically favorable configuration, showing the highest peak current and the smallest Δ*E*_p_. This is further confirmed by the quantitative comparison in Figure 3 and Table S4, where this composition reached an *I*_pa_ of 99.2 μA and a Δ*E*_p_ of 145.2 ± 3 mV after P-EC treatment—parameters consistent with quasi-reversible electron transfer.

**Figure 3:**
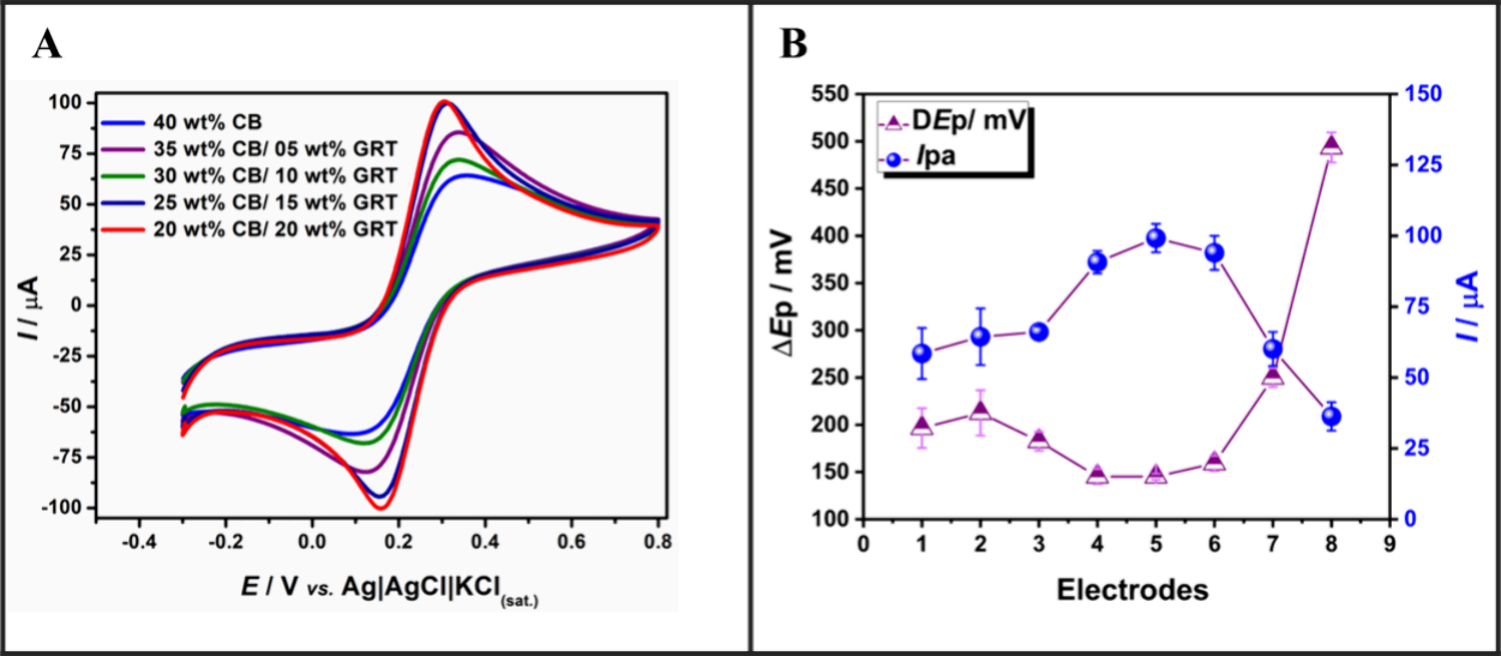
(A) Cyclic voltammograms recorded in [Fe­(CN)_6_]^4–/3-^ (1 mM in 0.1 M KCl) for CB/GRT/TPU electrodes after combined mechanical polishing and (electro)­chemical activation. (B) Correlation between anodic peak current (blue circles) and peak-to-peak separation (purple triangles) for electrodes 1 to 8. CV conditions: scan rate = 25 mV s^–1^; step potential = 5 mV.

In contrast, for compositions containing only CB and TPU (e.g., 40CB/0GRT) (Figure S8A), the P-EC treatment did not improve performance. On the contrary, electrochemical activation alone resulted in better-defined voltammograms and higher peak currents. This behavior suggests that mechanical polishing is only advantageous when graphite is present, likely due to the mechanical polishing exposing a higher percentage of edge planes within the graphite, leading to an increased electrochemical activity and a larger active surface area.^70–73^ When larger amounts of graphite are incorporated into the filament there is a sharp decrease in the performance for the P-EC electrodes. This is attributed to the structural continuity and stability of the conductive network, whereby synergy is obtained up to a point, after which there is insufficient CB to adequately link between the graphite flakes. This observation aligns with reports in literature, which explain that the combination of CB and GRT creates a hybrid conductive network with synergistic effects. Carbon black provides high-density local tunnelling paths due to its nanoscale agglomerates, while graphite contributes to macroscopic electron transport routes and mechanical stability.^59^ Moreover, GRT acts as a conductive bridge, reducing the gap between CB clusters and enhancing overall connectivity. These complementing structures allow polishing to effectively expose a more continuous and resilient surface for electron transfer, a result not achievable with CB alone.

Overall, the CV results highlight the importance of mixed filler composition and surface treatment strategies. When properly balanced, the combination of spherical CB and lamellar GRT enhances both local conductivity and network stability, which are critical parameters for achieving reproducible and efficient sensing platforms.

#### 3.3.2 Double-Layer Capacitance

The double layer capacitance (*C*_dl_) of the CB/GRT/TPU-based electrodes was next determined through cyclic voltammetry (CV) in 0.1 mol L^–1^ KCl, with scan rates ranging from 5 to 30 mV s^–1^. This approach allows estimation of the electrochemical active surface area by analyzing the capacitive current as a function of scan rate under non-Faradaic conditions. Figure 4 illustrates the CV profiles obtained for two electrode formulations: 40 wt% CB/TPU before and after (electro)­chemical activation (EC), and 20 wt% CB/20 wt% GRT/TPU before and after combined polishing and (electro)­chemical activation (P-EC). In both cases, activation treatments led to an increase in capacitive current, evidenced by broader voltammograms with greater enclosed areas. The EC-treated CB/TPU electrode (Figure 4A, purple lines) showed a clear enhancement in capacitive behavior compared to the untreated version (red lines), suggesting increased exposure of conductive domains and a more accessible surface for double-layer formation.

**Figure 4:**
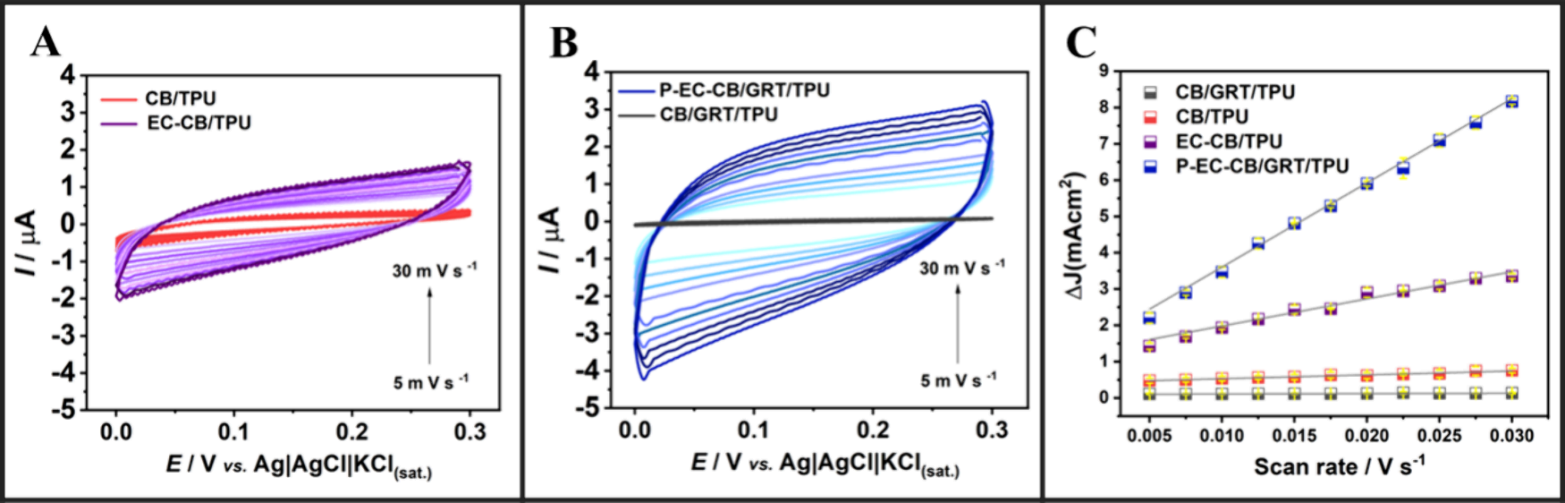
Determination of *C*_dl_ by cyclic voltammetry. (A, B) Cyclic voltammograms are recorded in 0.1 mol L^–1^KCl at scan rates from 5 to 30 mV s^–1^ for untreated and EC-treated 40 wt% CB/TPU electrodes (red and purple lines, respectively), and for untreated and P-EC-treated 20 wt% CB/20 wt% GRT/TPU electrodes (black and blue lines, respectively). (C) Corresponding Δ*J* vs scan rate plots. The slope of each linear fit represents the estimated *C*_dl_.

A more pronounced effect was observed for the 20 wt% CB/20 wt% GRT/TPU electrode (Figure 3B), where the P-EC-treated configuration (blue lines) exhibited substantially higher capacitive currents than the untreated one (black lines). This result highlights the combined effect of filler composition and surface treatment in optimizing electrode performance. The estimated *C*_dl_ values, obtained from the slopes of Δ*J* versus scan rate plots (Figure 4C), are summarized in Table S5. The P-EC-treated CB/GRT/TPU electrode exhibited the highest capacitance value, reaching 231.97 μF cm^–2^, which is more than three times higher than that of the EC-treated CB/TPU electrode (74.75 μF cm^–2^) and nearly 20 times higher than its untreated counterpart (10.65 μF cm^–2^). In contrast, the untreated CB/GRT/TPU electrode showed a very low capacitance (1.00 μF cm^–2^), reaffirming that surface modification is essential for maximizing electrochemical accessibility in composite systems. These findings demonstrate that the synergistic interaction between CB and GRT, when combined with effective surface conditioning via polishing and (electro)­chemical activation, is key to achieving a reactive surface. The increased *C*_dl_ values directly correlate with an improved electroactive area and confirm the suitability of this formulation for sensitive electroanalytical applications.

#### 3.3.3 Scan Rate Studies and Estimation of Electroactive Surface Area

To evaluate the electroactive surface area (*A*_e_) and electron transfer kinetics of the printed CB/GRT/TPU electrodes, cyclic voltammetry was performed at varying scan rates (5–500 mV s^–1^) using two benchmark redox systems: [Fe­(CN)_6_]^4–/3-^ and [Ru­(NH_3_)_6_]^3+^. These probes are widely used to characterize heterogeneous electron transfer (HET) processes, with the former being sensitive to surface fouling and the latter enabling more direct analysis of electroactive area. Utilization of both inner and outer sphere redox probes allows for insight into how the electrodes will perform against a wide range of molecules of interest. Scan rate studies (5–500 mV s^–1^) against the near-ideal outer-sphere redox probe [Ru­(NH_3_)_6_]^3+^ were performed, as this allows for accurate determination of the heterogeneous electron (charge) transfer rate constant (*k*_0_), electrochemical active area (*A*_e_), and provides a reliable benchmarking against other literature reports.^74,75^ The same experiments were run against the inner-sphere probe [Fe­(CN)_6_]^4–/3-^ to allow for complete comparison between the effect of surface modification techniques on the electrodes.

Figure 5 displays representative CVs for the EC-treated CB/TPU electrode and the P-EC-treated 20CB/20GRT/TPU electrode. Both systems exhibit increasing peak currents with increasing scan rates, while the linearity of the Randles–Ševčík plots (insets) confirms a diffusion-controlled process for both redox couples. Notably, the hybrid CB/GRT electrode outperformed the CB-only electrode, showing significantly higher peak currents and improved symmetry, especially for [Ru­(NH_3_)_6_]^3+^.

**Figure 5:**
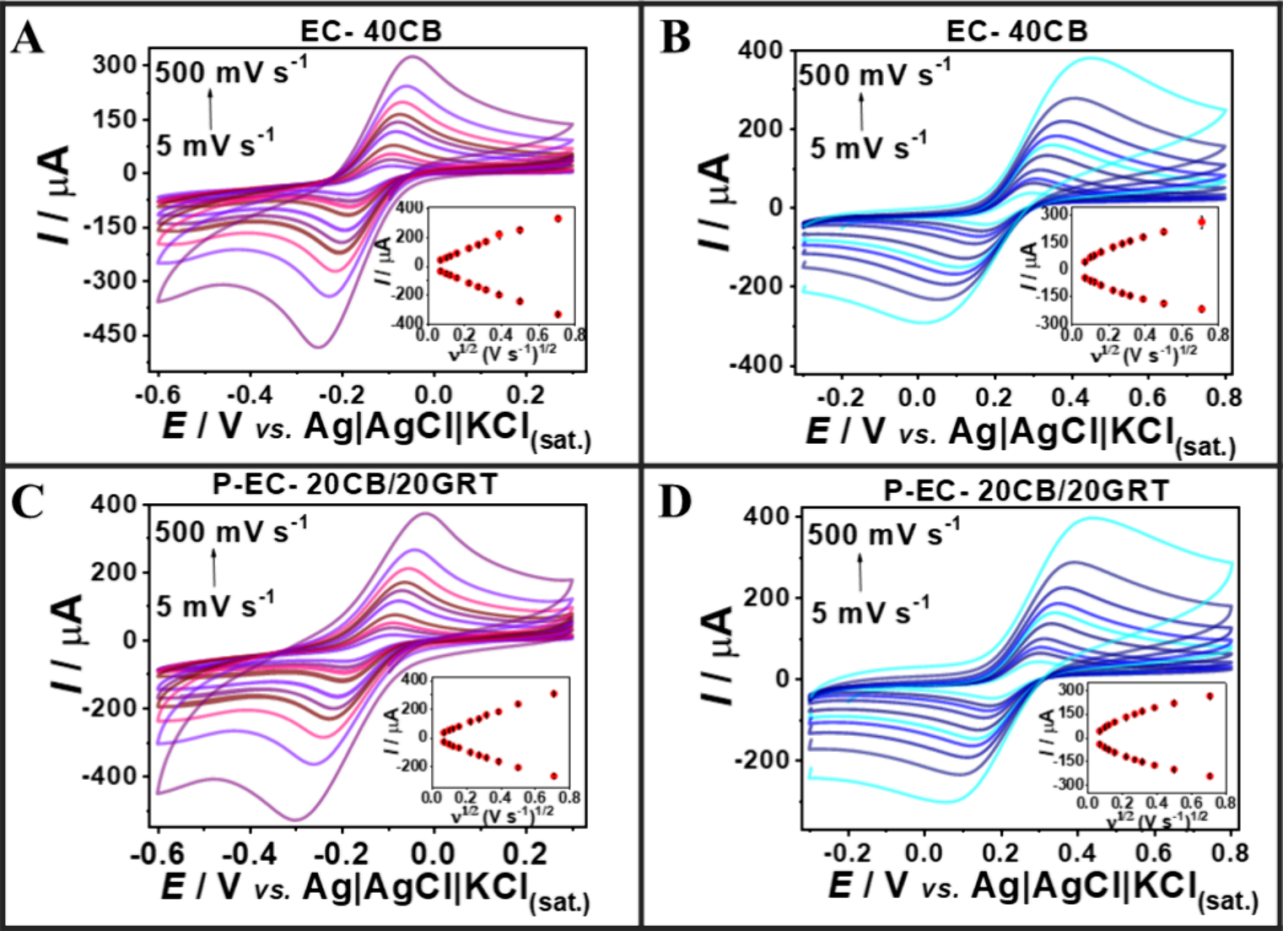
Cyclic voltammetry responses at different scan rates (5–500 mV s^–1^) for (A, B) 40 wt% CB/TPU electrode after (electro)­chemical activation and (C, D) 20 wt% CB/20 wt% GRT/TPU electrode after combined polishing and (electro)­chemical activation. Experiments were conducted in (B, D) 1 mmol L^–1^ [Fe­(CN)_6_]^4–/3-^ and (A, C) 1 mmol L^–1^ [Ru­(NH_3_)_6_]^3+^, both in 0.1 mol L^–1^ KCl. Insets: Randles–Ševčík plots.

This behavior is further elucidated by the broader data set presented in Figure S9, where different formulations and surface treatments were compared. The P-EC-treated electrodes generally exhibited more well-defined voltammograms, larger peak currents, and steeper slopes in the Randles–Ševčík plots than their EC-only counterparts. For example, the transition from EC to P-EC treatment in the 25CB/15GRT composition led to a pronounced increase in current (Figure S9B vs S9C and S9F vs S9G), highlighting the effect of polishing in removing surface barriers and exposing more conductive sites.

Quantitative values derived from these analyses are summarized in Table 2 and Table S4. For [Ru­(NH_3_)_6_]^3+^, the *A*_e_ of the P-EC-treated 20CB/20GRT electrode reached 0.60 ± 0.02 cm^2^, almost double the value observed for the same composition with EC alone (0.31 ± 0.02 cm^2^). This same trend was seen for electrodes tested against [Fe­(CN)_6_]^4–/3–^, where *A*_e_ of the P-EC-treated 20CB/20GRT electrode reached 0.77 ± 0.02 cm^2^, more than double the 0.31 ± 0.02 cm^2^ seen for EC-treated electrodes. Within [Fe­(CN)_6_]^4–/3–^, the P-EC treated electrodes also exhibited a higher *k*_0_ of 0.94 × 10^–3^ cm s^–1^ compared to their EC-treated counterparts with a *k*_0_ of 0.20 × 10^–3^ cm s^–1^, confirming improved interfacial kinetics for inner-sphere probes, a crucial parameter for electroanalytical applications.

**Table 2:** Electrochemically Active Surface Area (*A*_e_) and Heterogeneous Electron Transfer Rate Constants (*k*_0_) for [Fe­(CN)_6_]^4–/3-^ and [Ru­(NH_3_)_6_]^3+^ at CB/GRT/TPU Electrodes after (Electro)­chemical Activation (EC) and Combined Polishing with (Electro)­chemical Activation (P-EC)

**Electrode** (% CB/wt % GRT)	***A***_**e**_ **/cm** ^**2**^ **[Ru(NH**_**3**_ **)**_**6**_ **]** ^**3+**^	***k***_**0**_ **/×10** ^**–3**^ **cm s** ^**–1**^ **[Ru(NH**_**3**_ **)**_**6**_ **]** ^**3+**^	***A***_**e**_ **/cm** ^**2**^ **[Fe(CN)**_**6**_ **]** ^**4–/3–**^	***k***_**0**_ **/×10** ^**–3**^ **cm s** ^**–1**^ **[Fe(CN)**_**6**_ **]** ^**4–/3–**^
**EC-(40/00)**	0.68 ± 0.08	2.34 ± 0.32	0.75 ± 0.03	0.65 ± 0.08
**P-EC-(40/00)**	0.31 ± 0.08	1.64 ± 0.22	0.54 ± 0.05	0.48 ± 0.01
**EC-(35/05)**	0.48 ± 0.17	2.01 ± 0.06	0.57 ± 0.03	0.35 ± 0.05
**P-EC-(35/05)**	0.44 ± 0.04	1.67 ± 0.07	0.48 ± 0.09	0.49 ± 0.12
**EC-(30/10)**	0.54 ± 0.04	2.25 ± 0.13	0.41 ± 0.09	0.19 ± 0.08
**P-EC-(30/10)**	0.52 ± 0.00	1.67 ± 0.13	0.48 ± 0.02	0.71 ± 0.10
**EC-(25/15)**	0.31 ± 0.02	2.13 ± 0.16	0.32 ± 0.07	0.24 ± 0.07
**P-EC-(25/15)**	0.44 ± 0.02	1.71 ± 0.13	0.67 ± 0.05	0.95 ± 0.14
**EC-(20/20)**	0.31 ± 0.05	1.69 ± 0.06	0.31 ± 0.02	0.20 ± 0.03
**P-EC-(20/20)**	0.60 ± 0.02	1.38 ± 0.17	0.77 ± 0.02	0.94 ± 0.06

Interestingly, the effect of polishing was not always beneficial. As previously observed, for the CB-only electrode (40/00), P-EC treatment resulted in a decrease in *A*_e_ (from 0.75 ± 0.03 to 0.54 ± 0.05 cm^2^) and *k*_0_ (from 0.65 × 10^–3^ to 0.48 × 10^–3^ cm s^–1^). This suggests that, in the absence of graphite, polishing may expose fewer conductive domains or even disrupt existing ones. Conversely, when graphite is present in sufficient quantities (≥15 wt%), polishing enhances the continuity and accessibility of the conductive network and introduce increased amounts of reactive edge-plane sites. It is also important to note that by replacing 20 wt% of CB with graphite, the material cost of this filament is decreased from £111.47 per kg to £70.67 per kg, while actually improving the electrochemical performance. The ability to create high-performance parts while reducing expenditure makes this material extremely attractive for use in the healthcare sector.

Together, these findings demonstrate that both the electrode formulation and surface treatment play essential roles in determining electrochemical performance. The combination of CB and GRT enhances not only conductivity but also the electrode’s responsiveness to redox probes, particularly when optimized postprocessing protocols are applied. Given its enhanced surface activation and consistent electrochemical performance, the P-EC-CB/GRT/TPU electrode was selected for subsequent application studies involving UA detection using a wearable wristband platform. Its optimized structure and treatment make it a strong candidate for real-time sensing in flexible and miniaturized electrochemical devices.

### 3.4 Analytical Performance for Uric Acid Detection

After electrochemical characterization, the electroanalytical performance of the electrodes was tested to establish their potential for use within wearable sensors. To achieve this, UA was chosen as a target analyte due to its clinical relevance and presence within human sweat. The monitoring of UA levels is important, as its accumulation is linked to disorders such as gout, kidney impairment, and oxidative imbalance, making it a suitable target for sensing applications.^76,77^ Sweat, being a noninvasive and easily accessible biofluid, offers a convenient matrix for UA detection, especially when integrated into wearable sensor formats.^24,50^ Therefore, the performance of the electrodes was evaluated through the construction of two analytical curves corresponding to two different electrode systems. The first system employed commercial external Ag/AgCl/KCl (3 M) and nichrome wire as reference and counter electrodes, respectively, resulting in the first analytical curve, Figure 6A. In contrast, aiming at real-world applications and the development of wearable devices, the second system consisted of CB/GRT/TPU electrodes used as working, counter, and pseudoreference electrodes, forming a fully additively manufactured integrated setup that produced the second analytical curve, Figure 6B. In both cases, the same lollipop design as used in the previous section was used as the additively manufactured electrodes. Prior to measurements, the working electrodes were polished and electrochemically activated, then subsequently placed in an electrochemical cell with a final volume of 5 mL. The results are presented in Figure 6.

**Figure 6:**
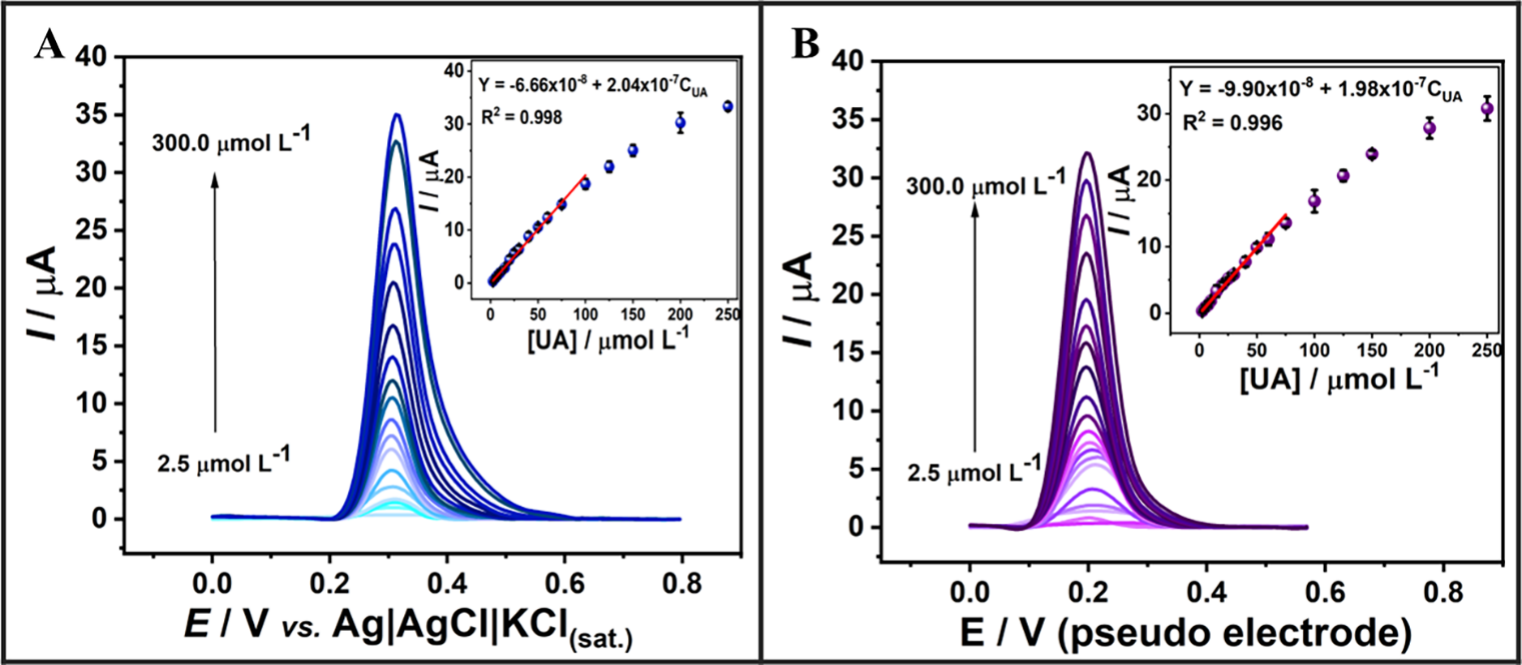
Differential pulse voltammetry for increasing concentrations of UA (2.5 to 300.0 μmol L^–1^) in 0.1 mol L^–1^ PBS recorded at the P-EC-CB/GRT/TPU electrode. (A) Measurements using an external Ag|AgCl reference and platinum counter electrode; (B) measurements using a fully printed three-electrode configuration (working, pseudoreference, and counter electrodes made from CB/GRT/TPU). Insets show the respective calibration curves with linear regression. DPV parameters: step potential = 5 mV, pulse amplitude = 50 mV.

For both configurations, well-defined anodic peaks were observed with increasing UA concentrations. Figures 6A and 6B present the differential pulse voltammograms of UA in 0.1 mol L^–1^ PBS, pH 7.4, for additions ranging from 2.5 to 100 μmol L^–1^, exhibiting excellent linearity (*R* ^2^ > 0.99), with the corresponding calibration plots shown as insets. Table S6 provides a comparison of the results obtained from the two calibration curves using the different electrode systems. It can be observed that the slope values obtained for both systems are similar, indicating that the sensitivity of the system is not significantly compromised when adopting the fully printed three-electrode configuration. Only the expected shift in the UA peak potential is observed when using the fully printed electrode’s setup. Furthermore, based on the calibration plots, the detectability of both systems was compared through the calculation of the limits of detection (LOD) and quantification (LOQ), which were determined using three and ten times the standard deviation of the intercept divided by the slope, respectively, for each calibration curve. As summarized in Table S6, LOD values of 1.3 and 1.7 μmol L^–1^ were obtained for the first and second systems, respectively, along with LOQ values of 4.5 and 5.5 μmol L^–1^. These results also confirm that detectability is not significantly affected when the fully printed three-electrode system is employed. In addition to the comparison between the two electrochemical configurations proposed in this study, the results obtained with the fully printed CB/GRT/TPU electrode system (the second system) were also compared with previous reports in the literature for UA quantification using various types of electrodes, including other additively manufactured configurations and devices applied to the detection of UA in artificial sweat samples (Table 3).

**Table 3:** Comparison of Electroanalytical Methods for UA Determination^a^

**Electrode**	**Sample**	**Technique**	**LOD** **(μmol L^–1^)**	**Linear range** **(μmol L^–1^)**	**Ref.**
CuNi-MOF@rGO	Sweat	AMP	9.09	10–1000	^78^
GCE/CTAB@SiO_2_@MWCNT-APTES	Human plasma and urine	DPV	4.5 × 10^–4^	1.5 × 10^–3^ to 1 × 10^–2^	^79^
Au@Ni-MOF (Ni(CH_3_ CO_2_)_2_)/SPCE	Human serum	DPV	0.19	0.5–1000	^80^
ITO-rGO-AuNPs	Milk, juice and urine	LSV	3.60	10–500	^81^
PMB/PLA-CB	Electrolyte (PBS 0.1 M)	DPV	9.61	50–1000	^82^
Sonogel-carbon electrode electrode modified with l-cysteine	Serum	SWV	10	10–100	^83^
P-EC-CB/GRT/TPU	Sweat	DPV	1.65	2.5–100	This work

a **Key**: CuNi-MOF@rGO: Nanomaterial composed of CuNi-metal-organic framework and reduced graphene oxide onto a polyimide substrate; GCE/CTAB@SiO_2_@MWCNT-APTES: composite consisting of multiwalled carbon nanotubes (MWCNTs) functionalized with 3-aminopropyltriethoxysilane (APTES), coated with a silica (SiO_2_) layer, and further modified with cetyltrimethylammonium bromide (CTAB) to enhance surface interaction and electrochemical performance; Au@Ni-MOF (Ni­(CH_3_CO_2_)_2_)/SPCE: screen-printed carbon electrode (SPCE) modified with gold-nanoparticle-decorated nickel-based metal-organic framework (Ni-MOF), synthesized using nickel acetate [Ni­(CH_3_CO_2_)_2_] as the metal precursor and 1,3,5-benzenetricarboxylic acid (H_3_BTC) as the organic ligand. AMP: Chronoamperometry; ITO-rGO-AuNPs: Indium tin oxide (ITO) based electrodes functionalized with reduced graphene oxide and gold nanoparticles; LSV: Linear Sweep Voltammetry; PMB/PLA-CB: Poly­(methylene blue) modified PLA-Carbon Black; DPV: Differential-pulse voltammetry; SWV: Square-wave voltammetry.

Among the studies reviewed, the method presented herein, based on a fully printed, in-lab fabricated electrode system, demonstrated superior detectability, as indicated by a lower limit of detection compared to the values reported in the consulted literature. Notably, the P-EC-CB/GRT/TPU electrodes developed herein require no further modification with additional materials, offering significant advantages in terms simplicity, low cost, and ease of fabrication, which are critical factors for scalable and practical applications.

### 3.5 Interference Study

In biological fluids such as sweat, compounds like urea, glucose, and tyrosine may coexist with UA and potentially interfere with its electrochemical detection. To evaluate the selectivity of the additively manufactured CB/GRT/TPU sensor, UA was tested at a fixed concentration of 10.0 μmol L^–1^ in the presence of these species at a 1:1 molar ratio (Figure S10). As shown, glucose and tyrosine had negligible influence on the UA signal, maintaining over 98% of the initial peak current response (98.8% and 99.9%, respectively). Urea caused a slightly more pronounced decrease in the UA current, reducing the response to 87.7%, yet still within acceptable limits (i.e., <15% deviation). These results confirm the high selectivity of the developed sensor for UA even in the presence of common endogenous interferents. This behavior is likely attributed to the favorable electrochemical affinity and electron transfer kinetics of UA at the activated surface of the printed electrodes. If necessary, small interferences such as that observed for urea could be effectively corrected by employing the standard-addition method during real-sample analysis.

### 3.6 Application in Sweat Samples Using Fully Additive Manufactured TPU Wristband

To complete the electroanalytical evaluation of the fully printed three-electrode P-EC-CB/GRT/TPU system, the quantification of UA was performed in artificial sweat. This represents a promising alternative for noninvasive UA monitoring, as changes in sweat UA levels may precede alterations in blood concentrations, thereby offering an early warning for medical intervention, such as in cases of renal dysfunction, enabling earlier diagnosis.^84,85^ Moreover, continuous and real-time monitoring of UA in sweat, achievable through wearable sensors, is particularly valuable for chronic patients, therapeutic drug monitoring, and lifestyle or dietary adjustments.

Given that the electrodes presented in this work exhibit not only excellent analytical performance but also key features such as flexibility, low cost, and biocompatibility, they are well-suited for wearable device applications. Therefore, the UA detection studies in artificial sweat samples were carried out by integrating the three printed electrodes into a flexible wristband, also fabricated using nonconductive TPU as detailed in the Experimental Section. For these measurements, a total volume of 750 μL of solution (electrolyte or sample) was added directly into the wristband’s sensing chamber. The wristband was positioned horizontally during the measurements, with electrical connections made via the underside of the device, as shown in Figure 7. It is important to highlight that integrating TPU-based electrodes onto a TPU-made wristband offers a significant advantage in creating a leak-proof device. Due to the inherent rubber-like properties of TPU, the material naturally seals any gaps formed during electrode placement, ensuring a tight and secure fit that prevents leakages (Figure 7B).

**Figure 7:**
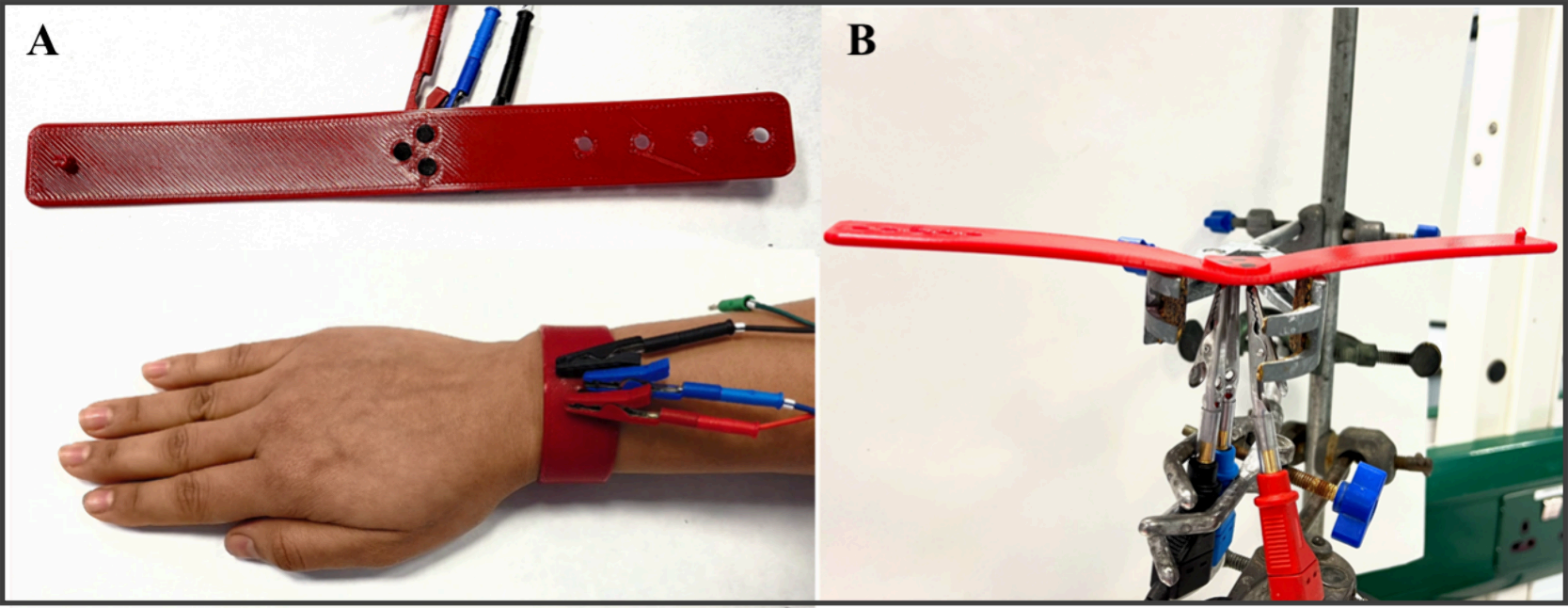
Photographs of the flexible wearable sensor device. (A) TPU-based wristband featuring integrated CB/GRT/TPU electrodes attached on the forearm with external connectors used for electrochemical measurements. (B) Side view of the device during the analytical procedure, illustrating the application of UA in artificial sweat for in situ detection.

For the addition and recovery studies, the sweat sample, diluted 1:1 with PBS, was spiked with two different UA concentration levels. The results obtained from the standard addition calibration are shown in Figure 8, while the spiked concentrations and corresponding recovery percentages are presented in Table 4.

**Figure 8:**
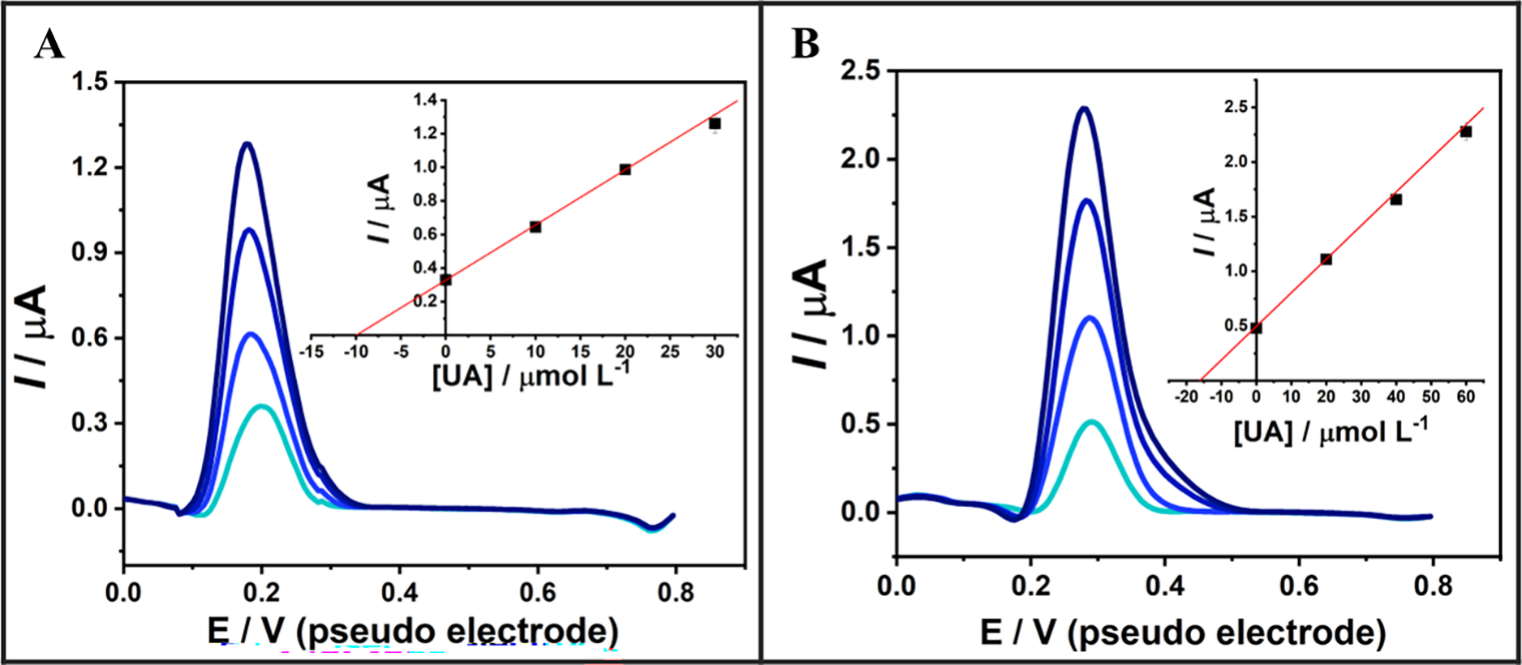
Determination of UA in artificial sweat using a wearable CB/GRT/TPU-based printed three-electrode system integrated into a flexible wristband. Detection was performed at two fortification levels: (A) 10 μmol L^–1^ and (B) 20 μmol L^–1^. DPV parameters: step potential = 5 mV; pulse amplitude = 50 mV.

**Table 4:** Results (Mean ± Standard Deviation) Obtained for the Determination of UA in Artificial Sweat Samples by DPV Using the CB/GRT/TPU-Based Sensor

**Sample**	**Spiked/** **μmol L^–1^**	**Found/** **μmol L^–1^**	Recovery/%
Sweat A	10.0	9.97 ± 0.80	99.7 ± 8.0
Sweat B	20.0	16.52 ± 0.17	82.6 ± 0.7

The results obtained show that the standard addition method applied produced a highly linear calibration curve, which was used alongside the spiked artificial sweat samples to calculate recovery values. UA exhibited excellent recoveries at both concentration levels, close to 100%, demonstrating the high accuracy of the system proposed in this study, even when employing three fully printed electrodes integrated into a novel wearable device. Importantly, when considering the material cost of the filament, the electrodes printed for the wristband sensor cost less than £0.01 per electrode, highlighting this technology’s potential to revolutionize wearable healthcare sensors.

Therefore, this study reports the development and successful application of flexible electrodes in two distinct configurations, fabricated using carbon black, graphite, and TPU. The electrodes exhibited outstanding analytical performance and were effectively integrated into a wearable device, enabling reliable detection of UA in artificial sweat. This approach demonstrates substantial potential for healthcare diagnostics and real-time monitoring, paving the way for the continuous detection and of a broad range of analytes in various biological and environmental matrices.

## 4 Conclusions

In this study, high-performance conductive filaments based on thermoplastic polyurethane (TPU) with varying ratios of carbon black (CB) and graphite (GRT) were successfully developed and evaluated for application in flexible electrochemical sensors. A systematic investigation of their physicochemical and electrochemical properties identified the 20 wt% CB and 20 wt% GRT formulation as the optimal composition, achieving an exceptional balance of electrical conductivity, mechanical flexibility, and electrochemical activity. Notably, this formulation also delivered a 45% reduction in material costs, underscoring its strong potential for scalable, cost-effective manufacturing of wearable sensing technologies.

Surface treatments played a critical role in enhancing sensor performance. While electrochemical activation (EC) improved all compositions, the combined treatment with mechanical polishing followed by EC (P-EC) yielded markedly superior results, particularly for GRT-containing formulations. Electrodes subjected to P-EC treatment exhibited significantly enhanced electrochemical characteristics, including the highest electroactive surface areas, faster electron transfer rates, and significantly enhanced double-layer capacitance (*C*_dl_). Specifically, the P-EC-CB/GRT/TPU electrode reached a *C*_dl_ of 231.9 μF cm^–2^, confirming its extensive surface activation and strong suitability for sensing applications.

This optimized electrode was then integrated into a fully printed, flexible wristband to evaluate its practical applicability in the detection of UA in artificial sweat, enabling noninvasive and on-body electrochemical sensing. Using differential pulse voltammetry, both the wristband-integrated system and the configuration with external electrodes demonstrated excellent analytical performance, with LOD values as low as 1.34 μmol L^–1^ and high linearity (*R* ^2^ > 0.99) across a broad UA concentration range. Importantly, the fully printed three-electrode configuration maintained comparable sensitivity to the system using external electrodes, validating the reliability of the printed platform. Recovery tests in artificial sweat confirmed the system’s viability, with values ranging from 82.6% to 99.7%, even in complex biological matrices. These results demonstrate the potential of the P-EC-CB/GRT/TPU electrode as a robust component for real-time UA monitoring in wearable formats, supporting early detection strategies for conditions such as kidney dysfunction and gout. Altogether, this work contributes a promising strategy for the additive manufacturing of flexible, low-cost, and efficient electrochemical sensors, advancing the field of wearable bioelectronics and personalized healthcare.

## Supplementary Material



## Notes

A summary of filament resistances and photographs of produced filaments and printed electrodes, TGA data, supplementary XPS data, contact angle data and supplementary electrochemical data (PDF)
