# Supplementary material for: Additive Manufacturing of Customized Flexible Wearable Sensors for Sweat Analysis with Bespoke, Low-Cost Conductive TPU
Source: ACS Meas Sci Au. 2025 Sep 26;5(6):842–56. doi: 10.1021/acsmeasuresciau.5c00095 (PMC12715732; doi:10.1021/acsmeasuresciau.5c00095)
Supplement: Supplementary file 1 [file tg5c00095_si_001.pdf]

Supporting Information for:

Additive manufacturing of customised flexible wearable sensors for sweat analysis with bespoke, low-cost conductive TPU

Mayane S. Carvalho[a,b‡], Elena Bernalte[a‡], Ana C. M. Oliveira[a,b], Eduardo M. Richter[b], Rodrigo A. A. Muñoz[b], Robert D. Crapnell[a], and Craig E. Banks[*a]

[a] *Faculty of Science and Engineering, Manchester Metropolitan University, Chester Street, M1 5GD, United Kingdom.*

[b] *Institute of Chemistry. Federal University of Uberlândia, 38400-902 Uberlândia, Minas Gerais, Brazil*

[‡]Indicates equal contribution

[*]To whom correspondence should be addressed.

1 E-mail: c.banks@mmu.ac.uk; Tel: +44(0)1612471196

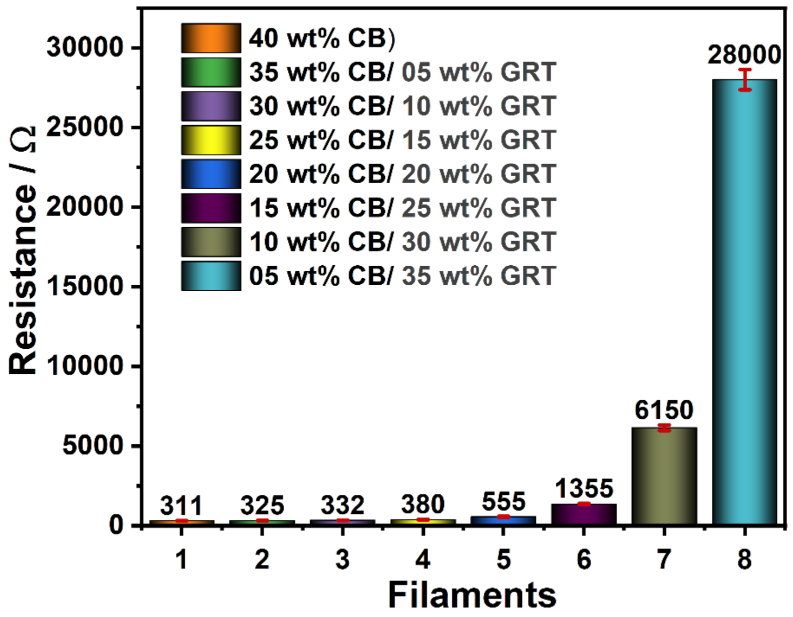

Figure S1. Electrical resistance of bespoke CB/GRT/TPU filaments as a function of carbon black (CB) and graphite (GRT) content. Resistance values refer to 10 cm of filament.

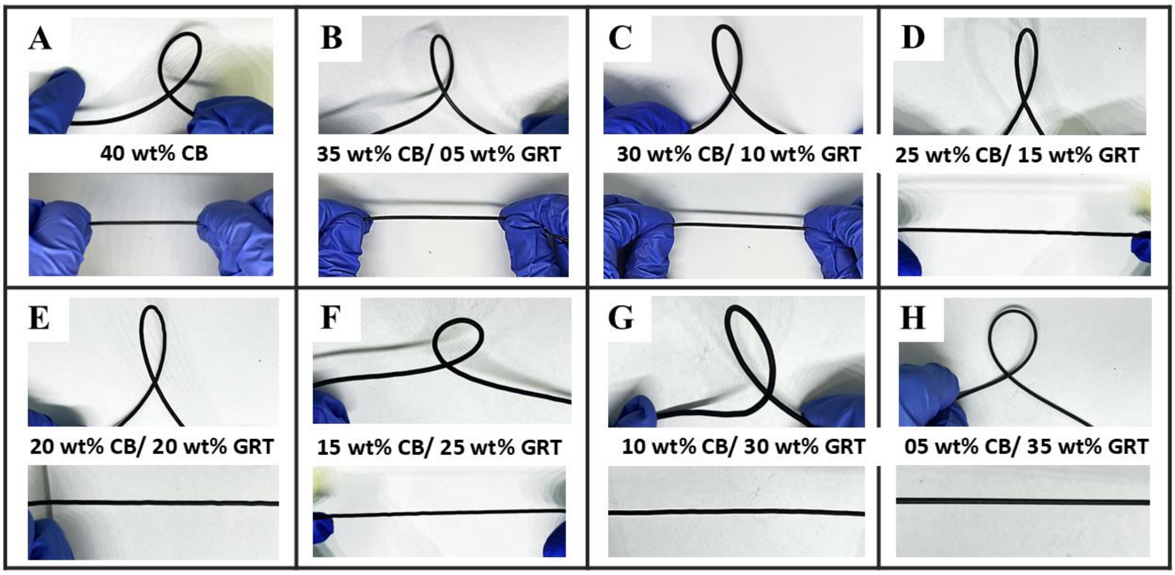

Figure S2. Visual assessment of flexibility and appearance of bespoke CB/GRT/TPU conductive filaments with varying carbon black (CB) and graphite (GRT) content. Filaments A–H correspond to formulations with increasing GRT content (and decreasing CB content), as indicated in each image.

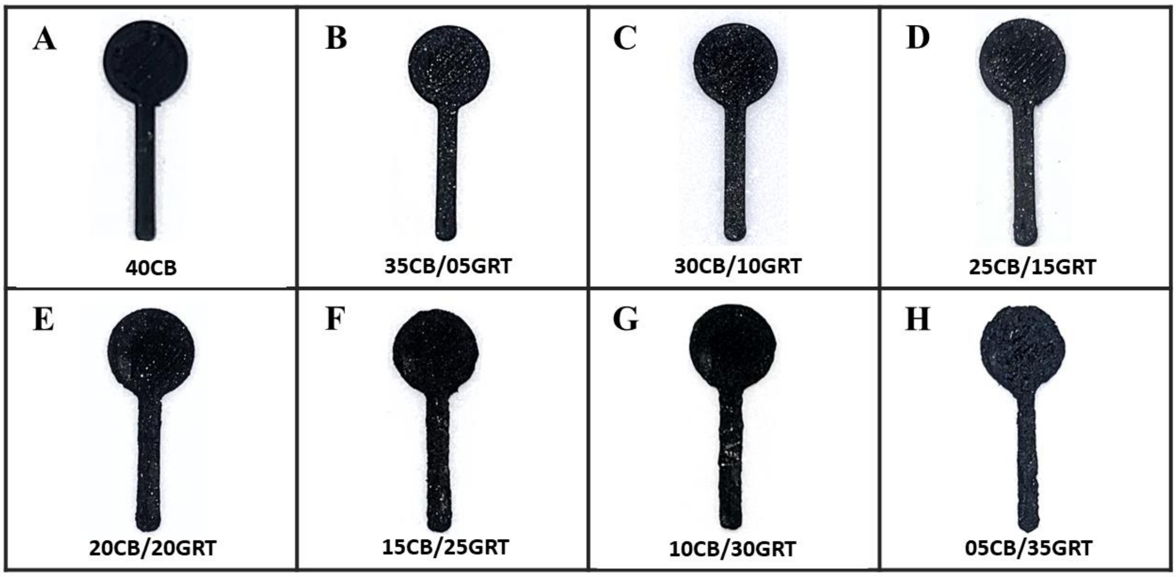

Figure S3. Visual appearance of as-printed CB/GRT/TPU electrodes. Images A–H correspond to different formulations with increasing graphite (GRT) and decreasing carbon black (CB) content.

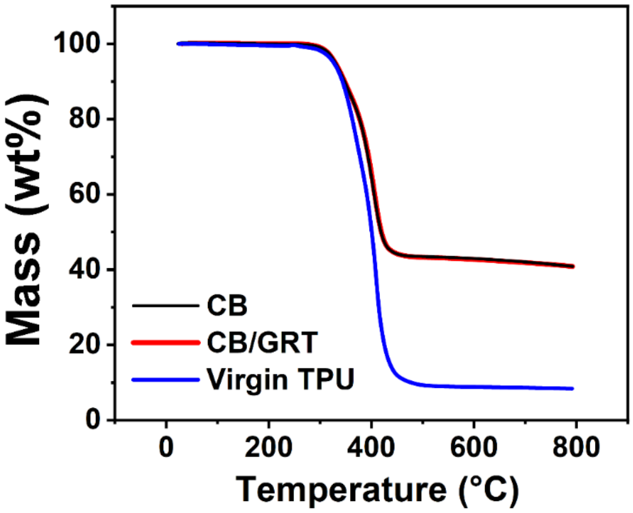

Figure S4. Comparison of thermogravimetric analysis (TGA) curves for virgin TPU and filaments containing CB and CB/GRT.

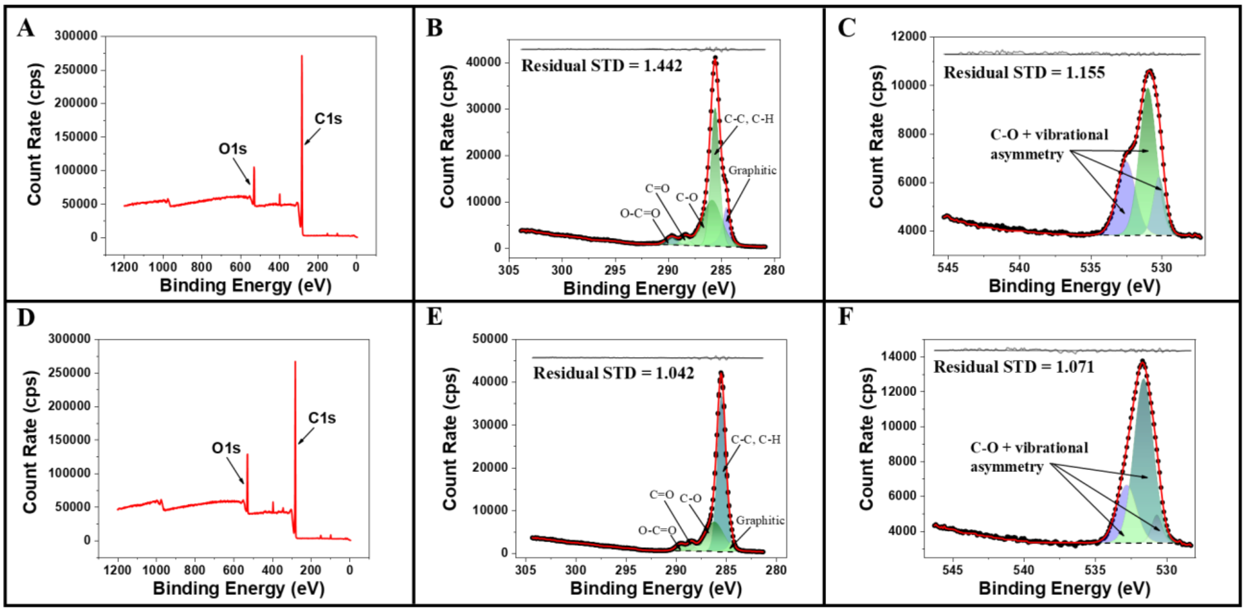

Figure S5. XPS data of the 40CB/TPU filament: **A)** Survey, **B)** C1s and **C)** O1s spectra of non-treated CB/TPU electrode. XPS data of the 20CB/20GRT electrode: **D)** Survey, **E)** C1s and **F)** O1s spectra of non-treated CB/GRT/TPU electrode.

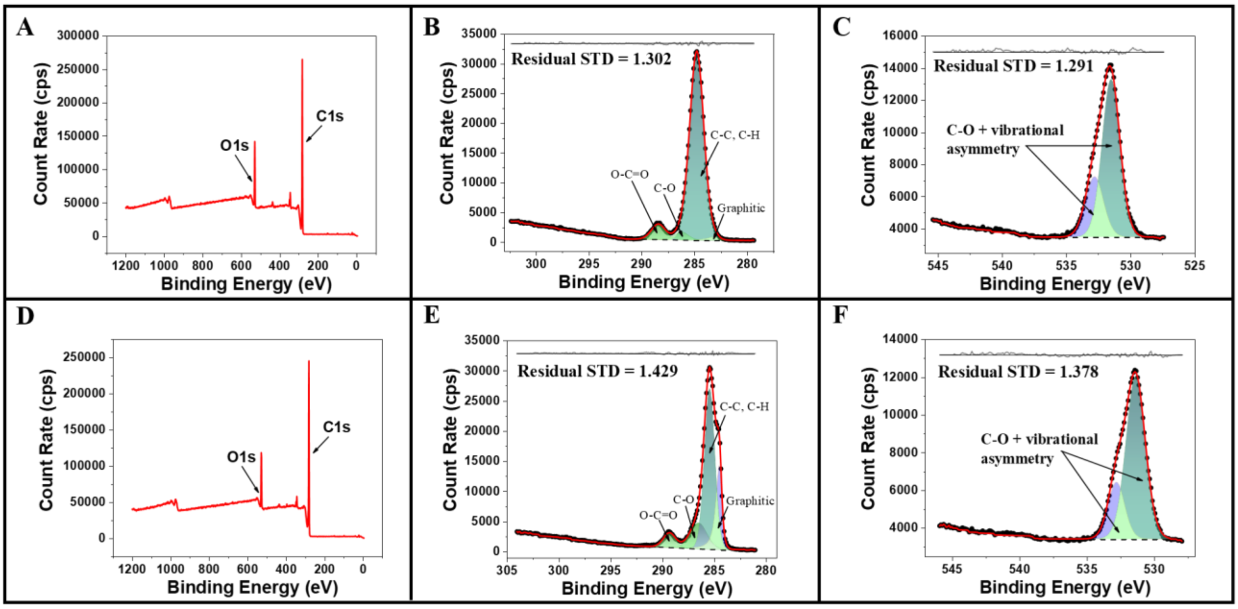

Figure S6. XPS data of the 40CB/TPU filament: **A)** Survey, **B)** C1s and **C)** O1s spectra of P-CB/TPU electrode. XPS data of the 20CB/20GRT electrode: **D)** Survey, **E)** C1s spectra and **F)** O1s spectra of P-CB/GRT/TPU electrode.

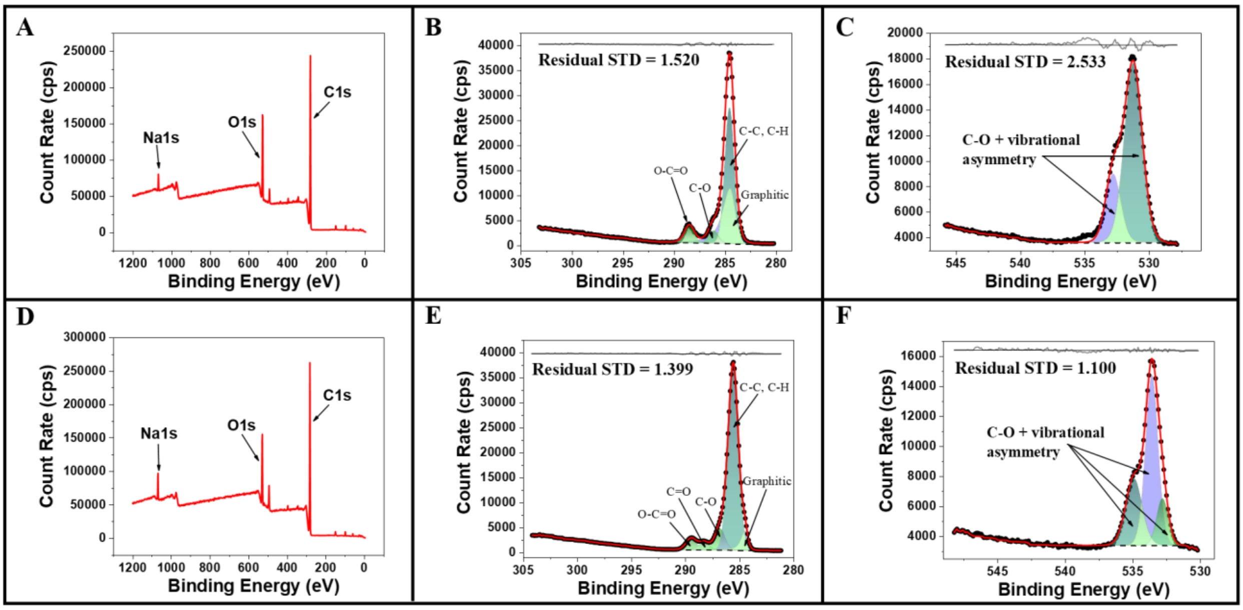

Figure S7. XPS data of the 40CB/TPU filament: **A)** Survey, **B)** C1s and **C)** O1s spectra of EC-CB/TPU electrode. XPS data of the 20CB/20GRT electrode: **D)** Survey, **E)** C1s and **F)** O1s spectra of EC-CB/GRT/TPU electrode.

Table S1. Concentration values of C 1s peaks (expressed in *atomic percent*, % at.) for 40CB electrodes with different surface treatments.

Sample	Graphitic	C-C	C-O	C=O	O-C=O
**40CB - CB/TPU**	12.01 ± 1.67	45.15 ± 2.91	36.35 ± 2.03	2.75 ± 0.38	3.73 ± 0.18
**40CB - P-CB/TPU**	0.89 ± 0.69	83.91 ± 3.10	7.89 ± 3.87	---	7.31 ± 0.41
**40CB - EC-CB/TPU**	35.67 ± 10.49	53.40 ± 10.21	4.74 ± 0.91	---	6.20 ± 0.74
**40CB - P-EC-CB/TPU**	0.78 ± 0.55	61.68 ± 12.75	30.96 ± 13.04	---	6.58 ± 0.81

Table S2. Concentration values of C 1s peaks for 20CB/20GRT electrodes with different surface treatments.

Sample	Graphitic	C-C	C-O	C=O	O-C=O
**20CB/20GRT - CB/GRT/TPU**	2.26 ± 0.76	64.04 ± 1.57	27.66 ± 1.22	2.48 ± 0.44	3.55 ± 0.18
**20CB/20GRT – P-CB/GRT/TPU**	17.86 ± 3.35	54.53 ± 4.38	21.33 ± 7.58	---	6.28 ± 0.18
**20CB/20GRT – EC-CB/GRT/TPU**	3.18 ± 2.77	73.83 ± 7.07	14.50 ± 8.93	3.36 ± 0.07	5.12 ± 0.44
**20CB/20GRT – P-EC-CB/GRT/TPU**	23.07 ± 1.83	62.80 ± 1.28	6.13 ± 0.51	1.8 ± 0.19	6.19 ± 0.33

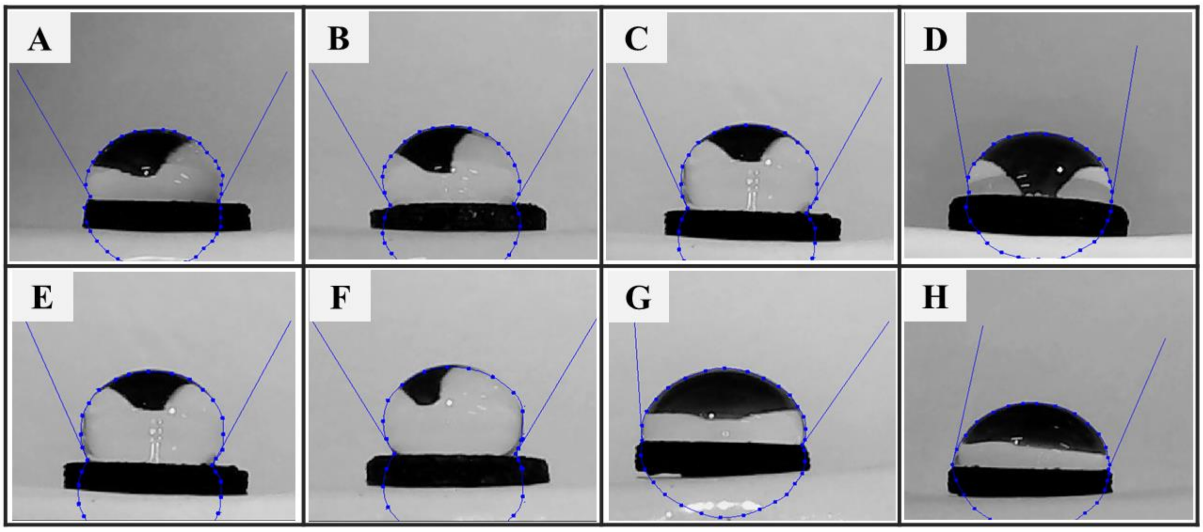

Figure S7. Contact angle images of bespoke CB/GRT/TPU-based electrodes. (**A**) Top and (**E**) bottom surfaces of the untreated electrode. (**B**) Top and (**F**) bottom surfaces after P treatment; (**C**) Top and (**G**) bottom surfaces after EC treatment; (**D**) Top and (**H**) bottom surfaces after P-EC treatment of the CB/GRT/TPU electrode.

Table S3. Mean contact angle values (± standard deviation) measured on the top and bottom surfaces of bespoke CB/GRT/TPU-based electrodes subjected to different surface treatments.

Electrode	Top side (º)	Bottom side (º)
**CB/GRT/TPU**	120 ± 2	114 ± 10
**P-CB/GRT/TPU**	120 ± 5	121 ± 3
**EC-CB/GRT/TPU**	118 ± 2	104 ± 6
**P-CB/GRT/TPU**	99 ± 4	107 ± 6

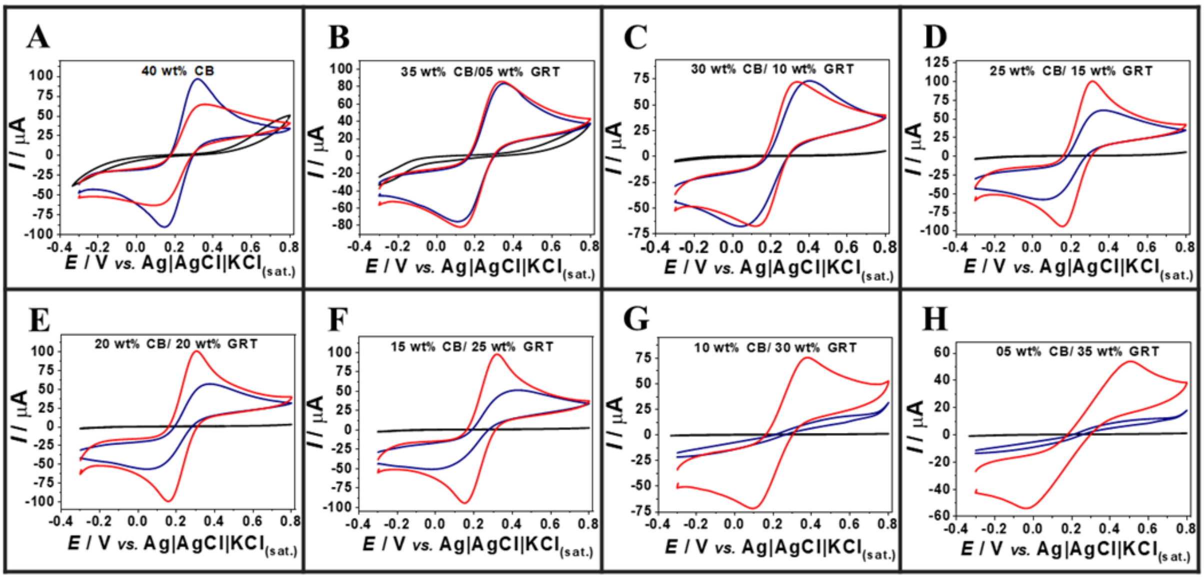

Figure S8. Cyclic voltammograms of bespoke CB/GRT/TPU electrodes (A–H) with increasing graphite content, recorded in 1 mmol L^{-1} $[Fe(CN)_6]^{3-/4-}$ solution. Black lines represent untreated (as-printed) electrodes, blue lines represent electrodes after electrochemical treatment, and red lines represent electrodes after combined polishing and electrochemical treatment. CV conditions: scan rate = 25 mV s^{-1}; step potential = 5 mV.

Table S4. Electrochemical parameters of CB/GRT/TPU electrodes after (electro)chemical activation and combined mechanical polishing with (electro)chemical activation. Parameters include anodic peak current, peak current ratio (Ipa/Ipc), and peak-to-peak separation (ΔEp), obtained in 1 mmol L^{-1} $[Fe(CN)_6]^{3-/4-}$.

**Electrode (% CB/% GRT)**	**(Electro)chemical activation**			**Polished and (electro)chemical activation**		
	Ipa/µA	Ipa/Ipc	ΔEp/mV	Ipa/µA	Ipa/Ipc	ΔEp/mV
**01 - (40/00)**	95.6	1.17	170 ± 11	58.5	1.16	196.5 ± 21
**02 - (35/05)**	75.3	1.25	244 ± 28	64.4	1.11	212.6 ± 24
**03 - (30/10)**	61.8	1.21	297 ± 44	66.1	1.12	182.6 ± 10
**04 - (25/15)**	42.5	1.25	298 ± 33	90.7	1.10	145.2 ± 8
**05 - (20/20)**	53.9	1.23	286 ± 23	99.2	1.09	145.2 ± 3
**06 - (15/25)**	43.7	1.28	313 ± 25	94.0	1.11	159.1 ± 8
**07 - (10/30)**	03.3	1.12	553 ± 17	60.1	1.05	249.9 ± 10
**08 - (05/35)**	02.9	1.71	404 ± 55	36.3	1.12	493.5 ± 16

Table S5. Double-layer capacitance (Cdl, µF cm^{-2}) values of 40 wt% CB/TPU and 20 wt% CB/ 20 wt% GRT/TPU electrodes, before and after mechanical polishing and/or electrochemical activation.

**Electrode**	**Cdl/ µF cm^{-2}**
**CB/TPU**	10.65 ± 0.98
**EC-CB/TPU**	74.75 ± 1.46
**CB/GRT/TPU**	1.00 ± 0.10
**P-EC-CB/GRT/TPU**	231.97 ± 9.76

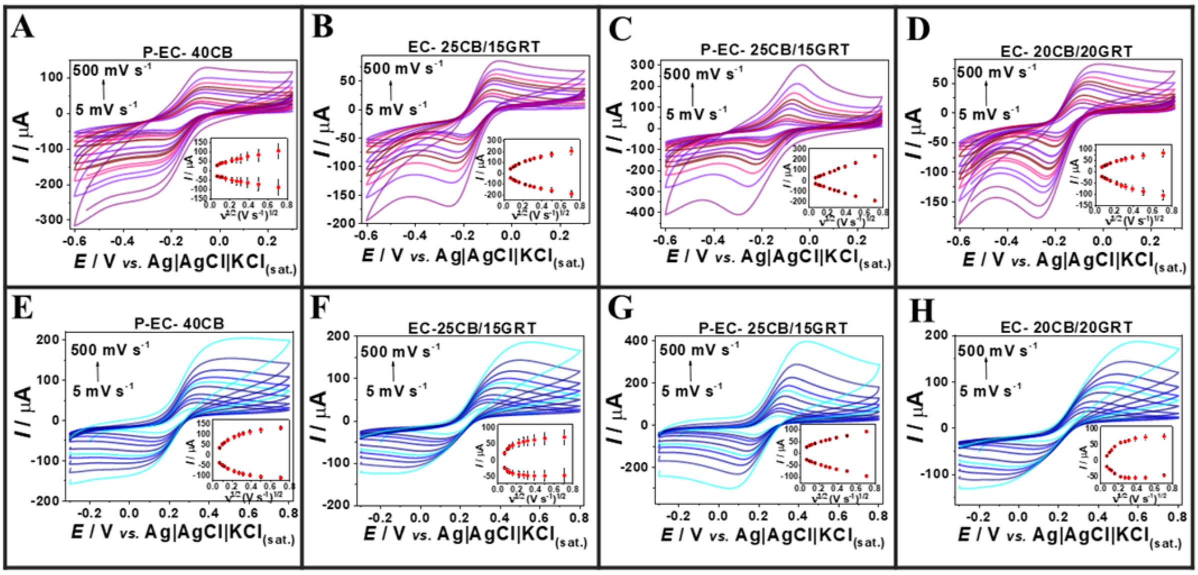

Figure S9. Cyclic voltammetry profiles of CB/GRT/TPU electrodes after (electro)chemical activation (EC) and combined polishing with (electro)chemical activation (P-EC), recorded at increasing scan rates (5 to 500 mV s^{-1}) in 1 mmol L^{-1} $[Ru(NH_3)_6]^{3+}$ (**A**–**D**) and 1 mmol L^{-1} $[Fe(CN)_6]^{3-/4-}$ (**E**–**H**), both in 0.1 mol L^{-1} KCl. Panels (**A**) and (**E**) correspond to P-EC-treated 40 wt% CB electrodes; panels (**B**), (**C**), (**F**), and (**G**) correspond to EC- and P-EC-treated 25/15 wt% CB/GRT electrodes; and panels (**D**) and (**H**) to EC-treated 20/20 wt% CB/GRT electrodes. Insets: Randles–Ševčík plots.

Table S6. Comparison of analytical performance for uric acid (UA) detection using external reference and counter electrodes and fully printed three -electrode CB/GRT/TPU systems.

Characteristics	External electrodes	Printed 3-electrode
**Linear range / µmol L^{-1}**	2.5 – 100.0	2.5 – 75.0
**R^2**	0.998	0.996
**Slope / µmol^{-1} L µA**	0.204 ± 0.004	0.198 ± 0.005
**Intercept / µA**	-0.067 ± 0.091	-0.099 ± 0.109
**LOD / µmol L^{-1}**	1.34	1.65
**LOQ / µmol L^{-1}**	4.46	5.51

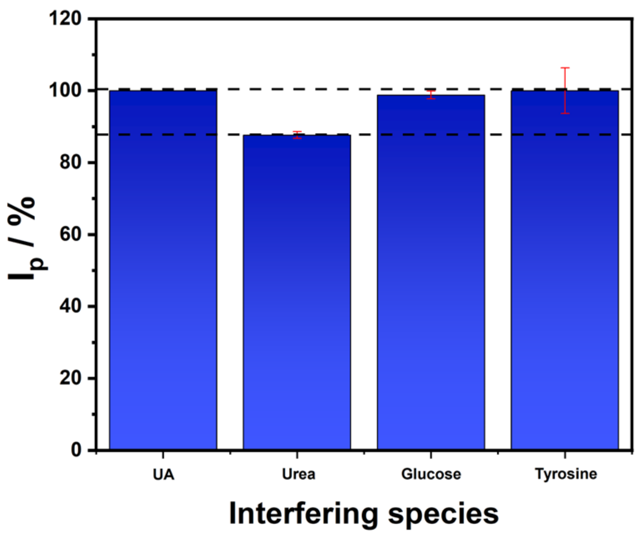

Figure S10. Selectivity assessment of the additive manufactured CB/GRT/TPU electrochemical sensor for uric acid (UA) detection (10.0 µmol L^{-1}) in the presence of potential interfering species (urea, glucose, and tyrosine) at a 1:1 molar ratio.
